# Longitudinal Monitoring of Parkinson's Disease in Different Ethnic Cohorts: The DodoNA and LONG-PD Study

**DOI:** 10.3389/fneur.2020.00548

**Published:** 2020-07-07

**Authors:** Katerina Markopoulou, Jan Aasly, Sun Ju Chung, Efthimios Dardiotis, Karin Wirdefeldt, Ashvini P. Premkumar, Bernadette Schoneburg, Ninith Kartha, Gary Wilk, Jun Wei, Kelly Claire Simon, Samuel Tideman, Alexander Epshteyn, Bryce Hadsell, Lisette Garduno, Anna Pham, Roberta Frigerio, Demetrius Maraganore

**Affiliations:** ^1^Department of Neurology, NorthShore University HealthSystem, Evanston, IL, United States; ^2^Department of Neuromedicine and Movement Science and Department of Neurology, St Olav's Hospital, Norwegian University of Science and Technology, Trondheim, Norway; ^3^Department of Neurology, Asan Medical Center, University of Ulsan College of Medicine, Seoul, South Korea; ^4^Department of Neurology, Laboratory of Neurogenetics, University of Thessaly, University Hospital of Larissa, Larissa, Greece; ^5^Department of Medical Epidemiology and Biostatistics, Karolinska Institutet, Stockholm, Sweden; ^6^Department of Clinical Neuroscience, Karolinska Institutet, Stockholm, Sweden; ^7^Health Information Technology, NorthShore University HealthSystem, Evanston, IL, United States; ^8^Program for Personalized Cancer Care, NorthShore University HealthSystem, Evanston, IL, United States; ^9^Department of Neurology, University of Florida College of Medicine, Gainesville, FL, United States

**Keywords:** longitudinal monitoring, Parkinson's disease, structured clinical documentation, motor symptoms, non-motor symptoms

## Abstract

**Background:** Different factors influence severity, progression, and outcomes in Parkinson's disease (PD). Lack of standardized clinical assessment limits comparison of outcomes and availability of well-characterized cohorts for collaborative studies.

**Methods:** Structured clinical documentation support (SCDS) was developed within the DNA Predictions to Improve Neurological Health (DodoNA) project to standardize clinical assessment and identify molecular predictors of disease progression. The Longitudinal Clinical and Genetic Study of Parkinson's Disease (LONG-PD) was launched within the Genetic Epidemiology of Parkinson's disease (GEoPD) consortium using a Research Electronic Data Capture (REDCap) format mirroring the DodoNA SCDS. Demographics, education, exposures, age at onset (AAO), Unified Parkinson's Disease Rating Scale (UPDRS) parts I-VI or Movement Disorders Society (MDS)–UPDRS, Montreal Cognitive Assessment (MoCA)/Short Test of Mental Status (STMS)/Mini Mental State Examination (MMSE), Geriatric Depression Scale (GDS), Epworth Sleepiness Scale (ESS), dopaminergic therapy, family history, nursing home placement, death and blood samples were collected. DodoNA participants (396) with 6 years of follow-up and 346 LONG-PD participants with up to 3 years of follow-up were analyzed using group-based trajectory modeling (GBTM) focused on: AAO, education, family history, MMSE/MoCA/STMS, UPDRS II-II, UPDRS-III tremor and bradykinesia sub-scores, Hoehn and Yahr staging (H&Y) stage, disease subtype, dopaminergic therapy, and presence of autonomic symptoms. The analysis was performed with either cohort as the training/test set.

**Results:** Patients are classified into slowly and rapidly progressing courses by AAO, MMSE score, H &Y stage, UPDRS-III tremor and bradykinesia sub-scores relatively early in the disease course. Late AAO and male sex assigned patients to the rapidly progressing group, whereas tremor to the slower progressing group. Classification is independent of which cohort serves as the training set. Frequencies of disease-causing variants in *LRRK2* and *GBA* were 1.89 and 2.96%, respectively.

**Conclusions:** Standardized clinical assessment provides accurate phenotypic characterization in pragmatic clinical settings. Trajectory analysis identified two different trajectories of disease progression and determinants of classification. Accurate phenotypic characterization is essential in interpreting genomic information that is generated within consortia, such as the GEoPD, formed to understand the genetic epidemiology of PD. Furthermore, the LONGPD study protocol has served as the prototype for collecting standardized phenotypic information at GEoPD sites. With genomic analysis, this will elucidate disease etiology and lead to targeted therapies that can improve disease outcomes.

## Introduction

Parkinson's disease (PD), the second most common neurodegenerative disease has an insidious onset and a long pre-symptomatic and symptomatic course. Four cardinal features that include resting tremor, bradykinesia, rigidity, and postural instability define the motor aspects of the disease. Different disease subtypes have been described including a tremor-predominant, akinetic/rigid predominant and mixed subtype (1). Non-motor features, including cognitive dysfunction, anosmia, anxiety, depression, sleep disorders, and autonomic dysfunction are also observed either alone or in varying combinations. Simuni et al. reported that for the Primary Progression Markers Initiative (PPMI) cohort, the higher baseline non-motor scores were associated with female sex and a more severe motor phenotype (2). Longitudinal increase in non-motor score severity was associated with older age and lower CSF aβ1–42 at baseline.

The temporal profile of the motor symptom appearance and progression is rather variable. A number of different patient cohorts have been followed longitudinally for different lengths of time and identified predictors of disease progression. Mollenhauer et al. analyzing the *De Novo* Parkinson (DeNOPA) cohort reported that baseline predictors of worse progression of motor symptoms included male sex, orthostatic blood pressure drop, diagnosis of coronary artery disease, arterial hypertension, elevated serum uric acid, and CSF neurofilament light chain (3). In the DeNOPA cohort, predictors of cognitive decline in PD were previous heavy alcohol abuse, current diagnoses of diabetes mellitus, arterial hypertension, elevated periodic limb movement index during sleep, decreased hippocampal volume by MRI, higher baseline levels of uric acid, C-reactive protein, high density lipoprotein (HDL) cholesterol, and glucose levels. In their cohort, risk markers for faster disease progression included cardiovascular risk factors, deregulated blood glucose, uric acid metabolism, and inflammation. Lawton et al. reported four clusters from the Tracking Parkinson's and Discovery cohorts: one with fast motor progression and symmetrical motor disease, poor olfaction, cognition, and postural hypotension; a second with mild motor and non-motor disease and intermediate motor progression; a third with severe motor disease, poor psychological well-being, and poor sleep with an intermediate motor progression; and a fourth with slow motor progression with tremor-dominant, unilateral disease (4). From the PPMI cohort, Aleksovski et al. reported that the postural instability gait disorder (PIGD) subtype was characterized by more severe disease manifestations at diagnosis, greater cognitive progression, and more frequent psychosis than tremor predominant patients (5). From the PPMI cohort, Latourelle et al. identified higher baseline MDS-UPDRS motor score, male sex, and increased age, as well as a novel Parkinson's disease-specific epistatic interaction, as indicative of faster motor progression (6). De Pablo-Fernandez et al. reported that the presence of autonomic symptoms contributed to a more rapid and severe disease course (7).

Comparing the findings of the different reported cohorts indicates partially overlapping clinical predictors. At the same time though, they reveal a variable clinical assessment. Here, we present an analysis of disease trajectory by GBTM in two large PD patient cohorts from five different countries followed at a routine clinical practice setting using identical clinical measures (8). We find that over an interval of 13 years, there are two trajectories, one with a more benign and another with a more severe disease progression. Patients can be reliably assigned to either group relatively early in their disease course.

## Methods

### Clinical Information

Two patient cohorts with PD were included in the study: (1) the DNA Predictions to Improve Neurological Health (DodoNA) cohort, which includes patients that are followed longitudinally in the Department of Neurology at NorthShore University HealthSystem in Evanston, Illinois and (2) the Longitudinal Clinical and Genetic Study of Parkinson's Disease (LONG-PD) cohort that includes PD patients enrolled by clinician investigators from Norway, Greece, South Korea, and Sweden. These investigators entered their clinical data through REDCap, a web-based database. The patient information that was submitted from the four different sites is referred to as the LONG-PD cohort in aggregate. The cohorts included both previously diagnosed and naïve patients. A copy of the study protocol is available in the Supplemental Information.

### The DodoNA Cohort

The goal of interpreting variation in DNA to predict neurological disease led to naming the NorthShore cohort as the “DodoNA” cohort after the Dodona oracle of ancient Greece. The content of the electronic medical record-based (EPIC systems) SCDS toolkit was developed through frequent movement disorder neurologist meetings aimed to reach a consensus on the essential data elements that conform to Best Practices in the treatment of PD, parkinsonism, or tremor patients, taking into consideration relevant literature and American Academy of Neurology (AAN) guidelines (9), and the International Consortium for Health Outcomes Measurement (ICHOM) guidelines (10). The criteria for which rating scales and score test measures to include in the toolkit were: (a) to obtain clinically relevant information in a standardized manner that can be performed at regular intervals; and (b) that the standardized assessment can be performed during an office visit within the time limitations that are imposed by a routine office visit. The toolkit content consists of discretized fields that record detailed information regarding initial and current symptoms, medication history and treatment response, and imaging results, as well as score test measures, including the Geriatric Depression Scale (GDS) (11), Epworth Sleepiness Scale (ESS) (12), United Parkinson's Disease Rating Scale (UPDRS) (13), Part I—Mentation, Behavior and Mood, UPDRS Part II—Activities of Daily Living (ADLs), UPDRS-Part III—Motor Score, UPDRS-IV—Complications of Therapy (COT), UPDRS-Part V—Hoehn and Yahr staging (H&Y), UPDRS-Part VI—Schwab & England Score (S&E), and the Short Test of Mental Status (STMS) (14) that are autoscored. For cognitive assessment, initially, the MoCA (Montreal Cognitive Assessment) (15) was used. However, due to licensing permissions, the STMS was subsequently used. Both scores (MoCA and STMS) can be converted to the Mini-Mental State Examination (MMSE) published nomograms (16) (unpublished data, with permission of Dr. Bradley Boeve, Mayo Clinic, Rochester, MN, USA), and therefore, all cognitive scores are recorded as the MMSE converted score.

The implementation of the toolkit has been cost effective, and the annual follow-up rates using the toolkit exceeded 85%.

### The LONG-PD Cohort

The clinical information for the LONG-PD cohort was entered by the neurologists from the four participant sites in the REDCap web-based tool designed for the electronic capture and sharing of data (http://project-redcap.org/). NorthShore built a REDCap form mirroring the DodoNA SCDS toolkit. A working group refined the form and defined required fields for all sites. The finalized form was presented at the annual meeting of the Genetic Epidemiology of Parkinson's Disease (GEoPD) Consortium in Vancouver, Canada (2015). All of the teams (DodoNA project, LONG-PD) are members of GEoPD. The REDCap format was chosen because it provides an easily accessible Interface for collecting and validating data, as well as automated data export to statistical packages in a secure, de-identified manner (SPSS, SAS, Stata, R).

### Data Treatment

Subjects were excluded that experienced onset of symptoms 10 years or more prior to their initial visit or that had less than two valid visits (at 1 year or greater intervals). Prior to assessment, subject scores were assumed to be unknown, and the study was limited to a 13-year period covering all patient visits in the cohort. Missing motor assessment scores were imputed as zero for calculation of patient tremor and bradykinesia sub-scores.

### Statistical Analysis

**Figure 1:**
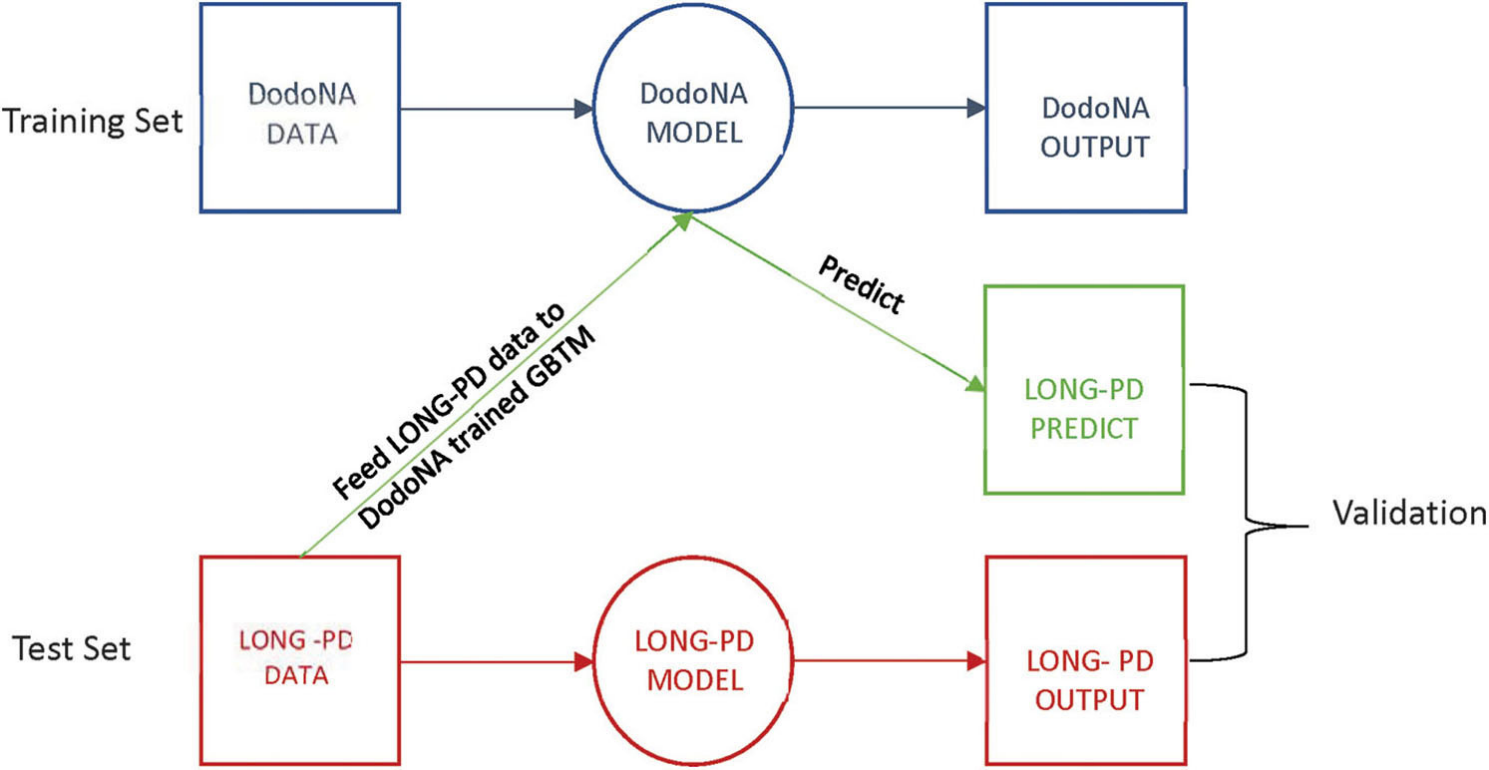
Group-based Trajectory Modeling flowchart.

Group-based trajectory modeling (GBTM) was applied to identify latent subgroups within the patient cohorts, given their covariates and assessment scores over time (17, 18). Assessment scores were taken on an annual basis during initial and annual follow-up visits. GBTM assigns individuals to separate latent subgroups with posterior probabilities over time and regression parameters to define the trajectory of those subgroups. The DodoNA cohort data were used as the training set and the LONG-PD data as the test set. The test set data were entered into the DodoNA model, and the output was compared to the LONG-PD test set for validation. This approach is illustrated in Figure 1.

Trajectories were calculated based on the year of the reported initial symptom when the patient is seen for the first time in the movement disorder clinic, thus extending the trajectory duration to a maximum of 13 years that included at least 5 years of follow-up for the DodoNA cohort and 3 years of annual follow-up for the LONG-PD cohort. This choice to include the interval from the reported initial motor symptom allows a more accurate assessment of the disease course as often the patients come to the clinic at different points in the disease process.

We tested models with one to two subgroups using either constant or linear terms. The best-fitting results were selected using the lowest Bayesian information criterion (BIC) value. We used fixed covariates including patient gender, age of onset of symptoms, positive family history of PD with multiple-member instances, tremor predominance, presence of autonomic symptoms (orthostatism, urinary incontinence, constipation) individually and in combination, levodopa therapy, dopaminergic therapy, and years of education. Each of the fixed covariates was then measured across assigned subgroups to determine group membership totals and statistical significance across subgroups (Wilcoxon rank sum test for continuous variables: age of onset, years of education; Pearson's chi-squared test for count data: all other covariates).

Latent subgroup classes in GBTM cannot be externally validated. However, we attempted to validate whether GBTMs trained on the DodoNA cohort would be predictive of patient subgroup membership in the LONG-PD cohort. To do this, we trained GBTMs on the DodoNA patient cohort (the “training” set), and using their covariate estimates with respect to baseline, predicted subgroup membership when applied to LONG-PD patients for each sub-score. As a validation measure, we separately applied GBTM to the LONG-PD cohort using the same external model parameters (number of subgroups to stratify patients, shape of subgroup trajectories) and assumed these results to be the ground truth “test” set. We validated the overall results of the prediction and test sets using confusion matrices and statistics to assess the GBTM predictive value. The GBTM analysis was also performed in reverse with the LONG PD cohort as the training set and the DoDoNA cohort as the test set.

All data were analyzed using STATA/IC 16.0 using the PROC TRAJ package, and the significance level was set at 0.05.

## Results

### Statistical Analysis

#### Assignment of Patients to Different Disease Trajectories Based on Individual Clinical Scores

**Figure 2:**
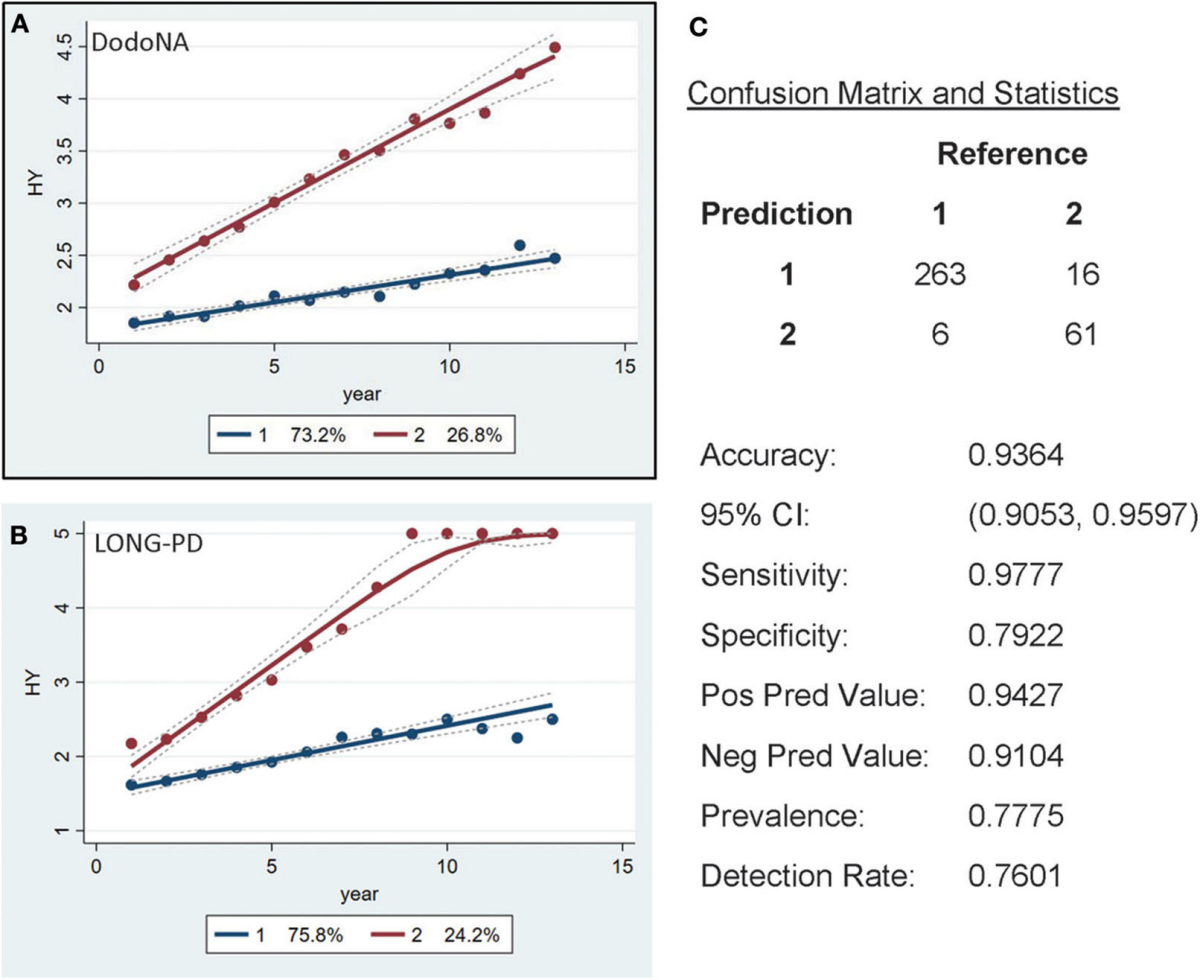
Hoehn and Yahr (H&Y) stage groups in the DNA Predictions to Improve Neurological Health (DodoNA) and Longitudinal Clinical and Genetic Study of Parkinson's Disease (LONG-PD) cohorts. **(A)** The model trained on DodoNA data (training set). **(B)** The model trained on the LONG-PD data (test set). **(C)** The validation for the LONG-PD prediction trained on NS data against the test set.

Individual clinical parameters were assessed for their effect on disease trajectory: With each clinical score with the exception of the tremor sub-score, two separate trajectories are clearly identified: one with a slower and less severe and one with a more rapid and more severe trajectory: for the H&Y stage (UPDRS-V) the group with a slower progression includes 73.2% of the cohort (Figure 2A). This is also observed in the LONG-PD cohort (Figure 2B) for 75.8% of the cohort. The validation for the LONG-PD prediction trained on the DodoNA test set is shown in Figure 2C with a sensitivity of 0.9777 and a specificity of 0.7922.

**Figure 3:**
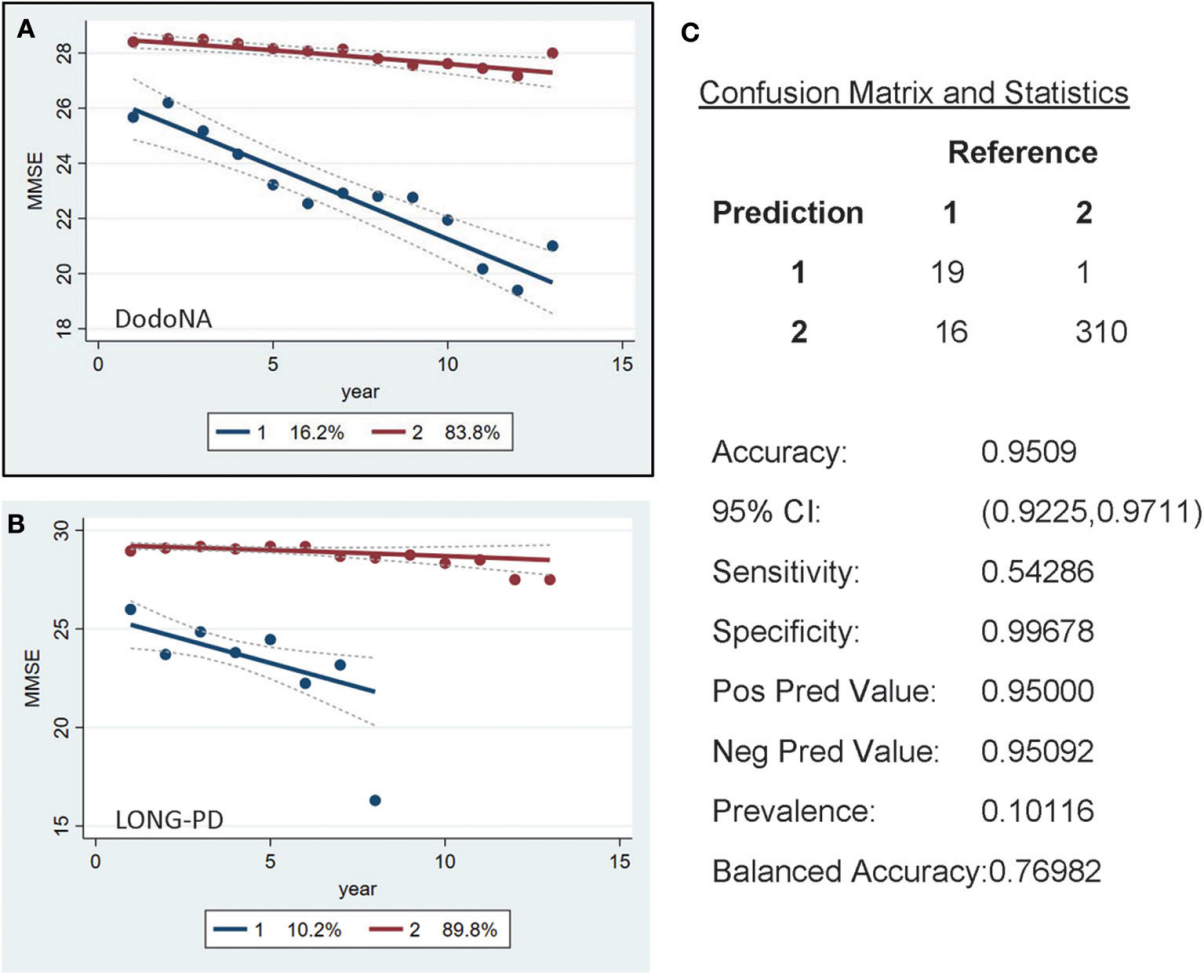
Mini-Mental State Examination (MMSE) score groups in the DodoNA and LONG-PD cohorts. **(A)** The model trained on DodoNA data (training set). **(B)** The model trained on the LONG-PD data (test set). **(C)** The validation for the LONG-PD prediction trained on NS data against the test set.

For the MMSE scores, a similar separation is seen with the larger subset [83.8% in the DodoNA cohort (Figure 3A) and 89.8% in the LONG-PD cohort (Figure 3B)] having a slower progression. The validation for the LONG-PD prediction trained on the DodoNA test set is shown in Figure 3C with a lower sensitivity of 0.54286 and a specificity of 0.99678. The apparent improvement of the MMSE scores, Figure 3B probably reflects the smaller sample size of the LONG-PD cohort.

**Figure 4:**
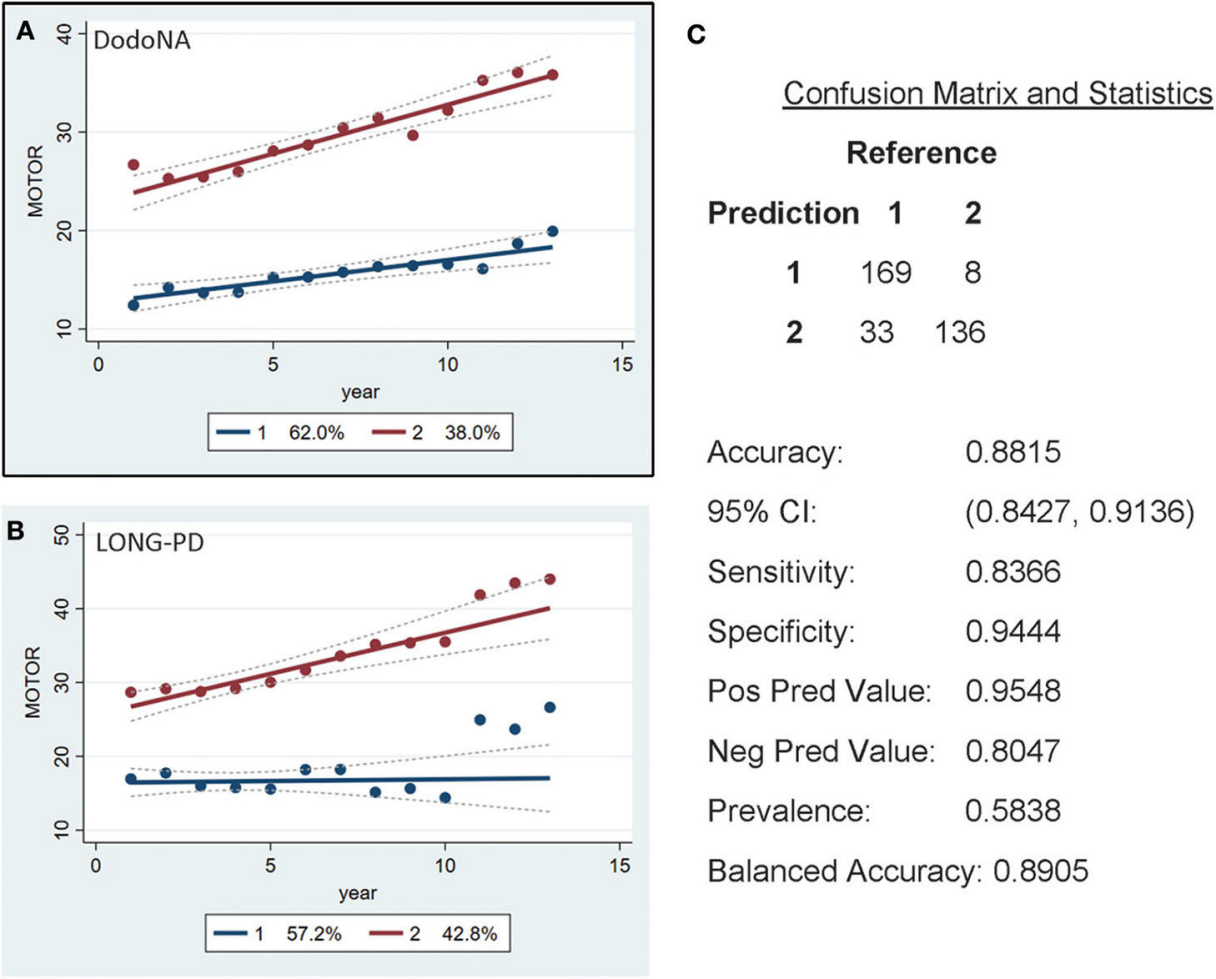
Motor Score groups in the DodoNA and LONG-PD cohorts. **(A)** The model trained on DodoNA data (training set). **(B)** The model trained on the LONG-PD data (test set). **(C)** The validation for the LONG-PD prediction trained on NS data against the test set.

For the UPDRS-III score, two groups are identified, with the slower progression group including 62% of the DodoNA cohort (Figure 4A) and 57.2% of the LONG-PD cohort (Figure 4B). The separation of the two trajectories appears less clear in the LONG-PD cohort, possibly reflecting treatment effects and shorter duration of follow-up. The validation for the LONG-PD prediction trained on the DodoNA test set is shown in Figure 4C with a sensitivity of 0.8366 and a specificity of 0.9444.

**Figure 5:**
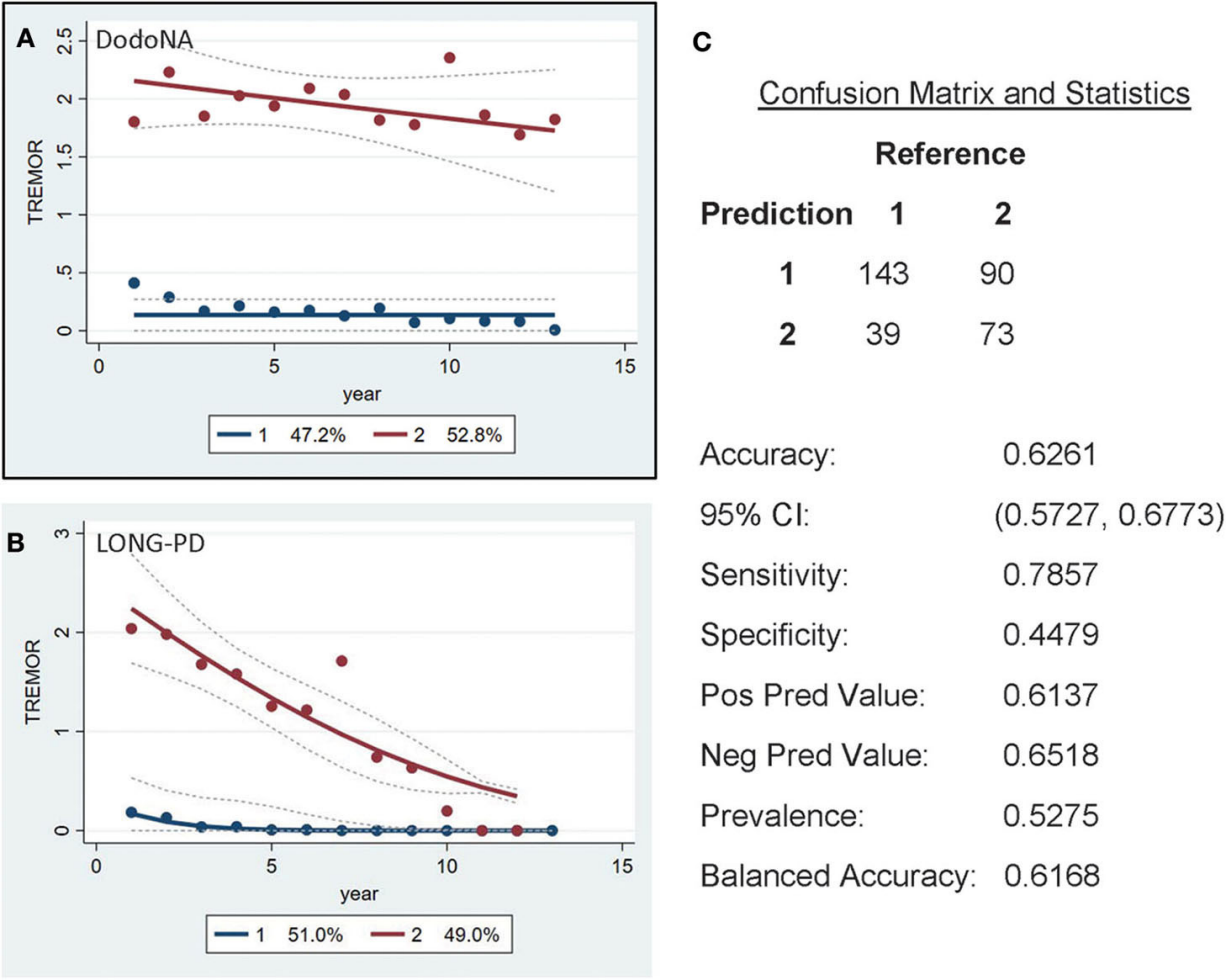
Unified Parkinson's Disease Rating Scale (UPDRS) (UPDRS)-III Tremor sub-score groups in the DodoNA and LONG-PD cohorts. **(A)** The model trained on DodoNA data (training set). **(B)** The model trained on the LONG-PD data (test set). **(C)** The validation for the LONG-PD prediction trained on NS data against the test set.

For the tremor sub-score of UPDRS-III, two groups are again identified: the slower progression group of the DodoNA cohort including 47.2% (Figure 5A) and the LONG-PD cohort 51% (Figure 5B). The validation for the LONG-PD prediction trained on the DodoNA test set is shown in Figure 5C with a sensitivity of 0.7857 and a specificity of 0.4479. The lower specificity that likely reflects the presence of tremor may not accurately reflect disease severity as it may be more sensitive to treatment effects.

**Figure 6:**
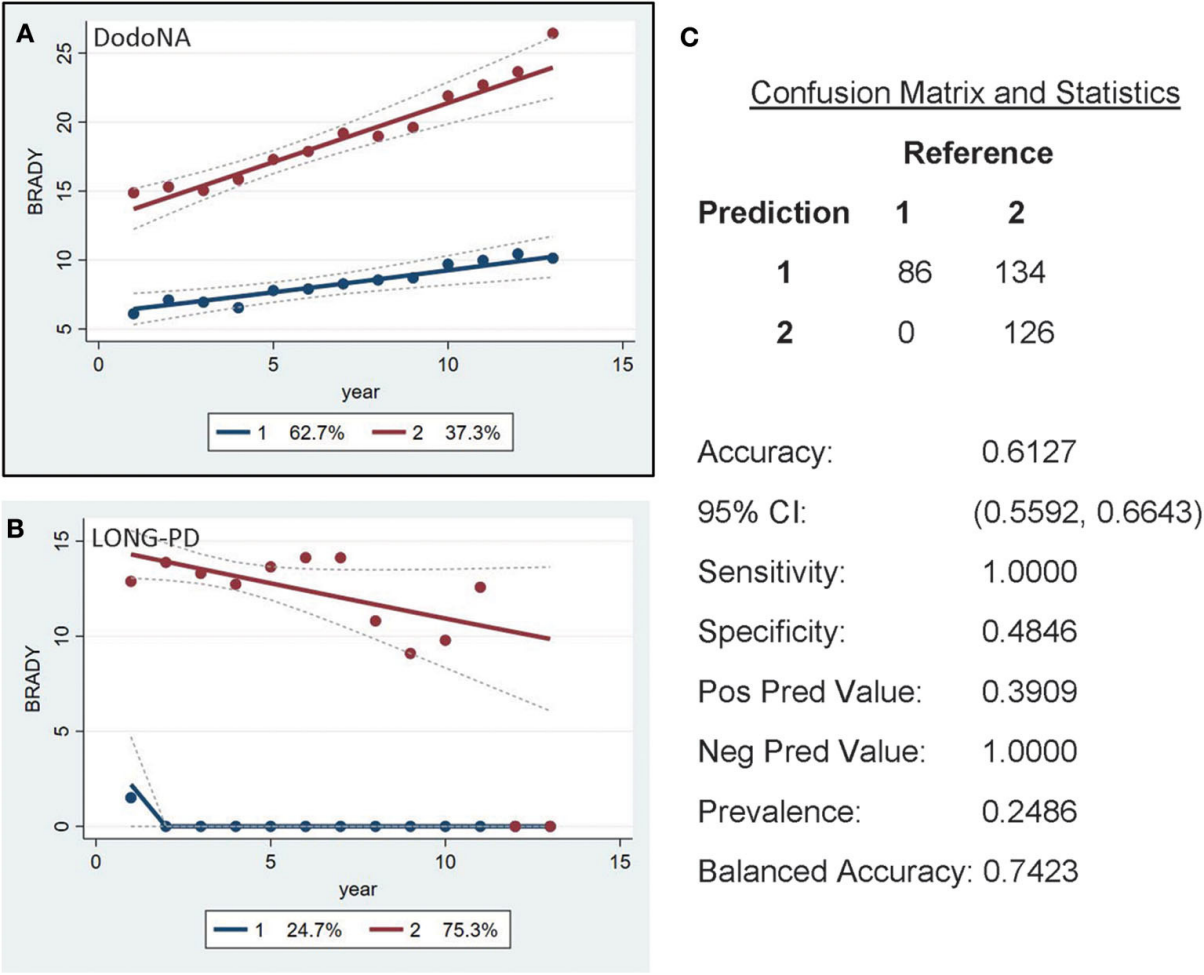
UPDRS-III Bradykinesia sub-score in the DodoNA and LONG-PD cohorts. **(A)** The model trained on DodoNA data (training set). **(B)** The model trained on the LONG-PD data (test set). **(C)** The validation for the LONG-PD prediction trained on NS data against the test set.

For the bradykinesia sub-score of UPDRS-III, two groups are again identified: the slower progression group of DodoNA cohort including 62.7% (Figure 6A) and the LONG-PD cohort including 24.7% (Figure 6B). The validation for the LONG-PD prediction trained on the DodoNA test set is shown in Figure 6C with a sensitivity of 1.000 and a specificity of 0.4648. The lower specificity likely indicated that sub-scores may not accurately reflect disease severity, as they only represent separate cardinal features and do not assess rigidity and postural instability.

**Figure 7:**
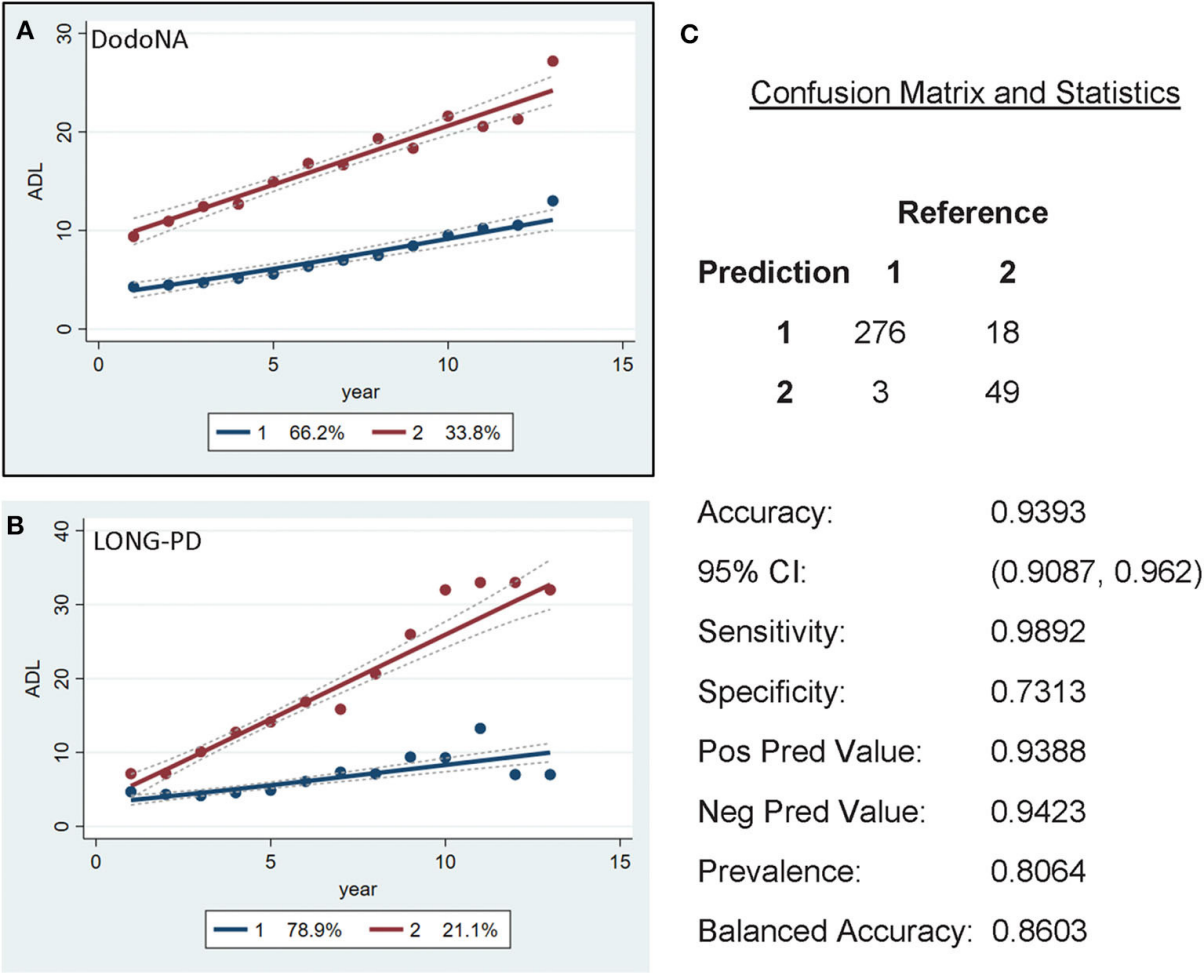
Activities of Daily Living (ADL) scores in the DodoNA **(A)** and LONG-PD **(B)** cohorts. **(C)** The validation for the LONG-PD prediction trained on NS data against the test set.

The UPDRS-II ADL score separates patients in two different trajectories, with 66.2% of the DodoNA cohort (Figure 7A) and 78.9% of the LONG-PD cohort (Figure 7B) showing a slow trajectory. The validation for the LONG-PD prediction trained on the DodoNA test set is shown in Figure 7C with a sensitivity of 0.9892 and a specificity of 0.7313. It is important to note that the UPDRS-II score reflects historical information and subject to a subjective assessment.

**Figure 8:**
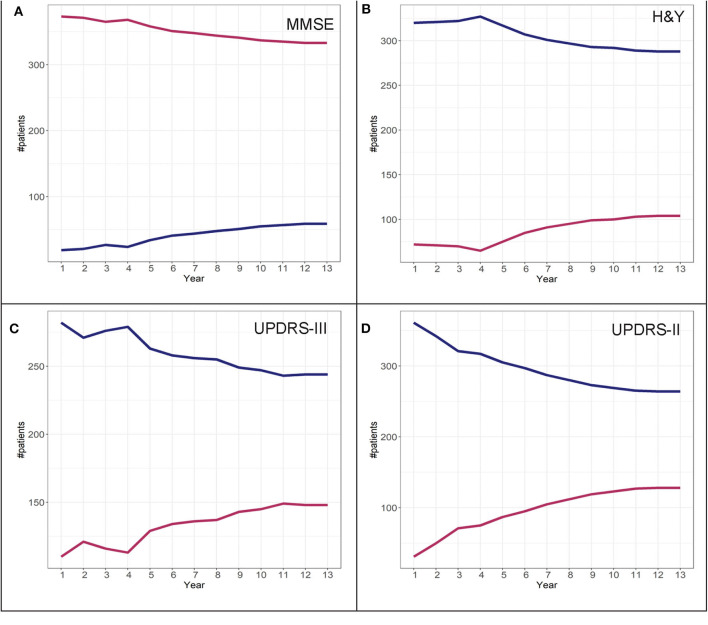
Convergence scores for MMSE **(A)**, H&Y stage **(B)**, UPDRS-III **(C)**, and UPDRS-II **(D)** for the DodoNA cohort.

**Figure 9:**
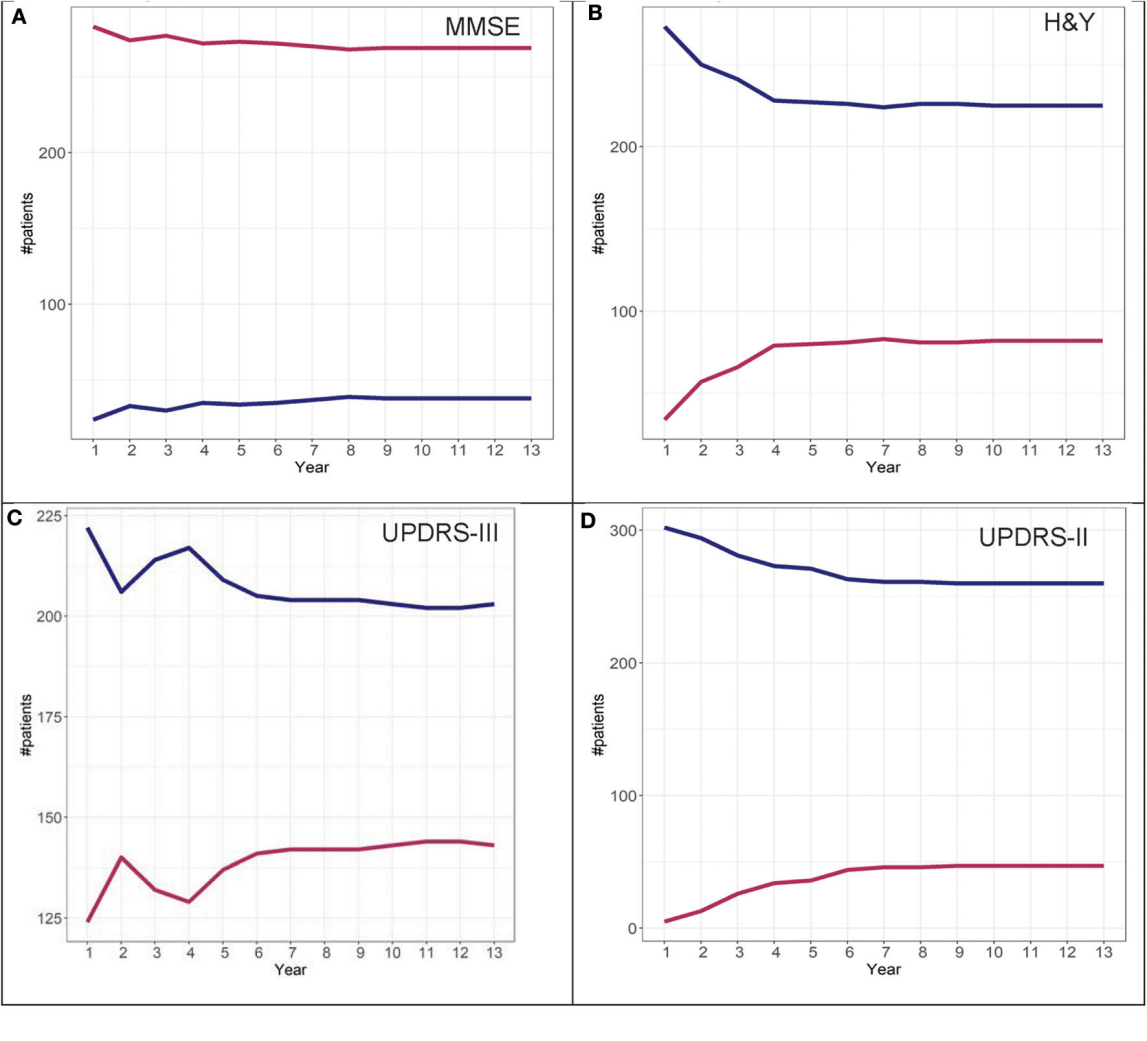
Convergence scores for MMSE **(A)**, H&Y stage **(B)**, UPDRS-III **(C)**, and UPDRS-II **(D)** for the LONG-PD cohort.

To determine adherence to a particular group identified in the GBTM, convergence graphs were generated based on the assumption that the group assignment at year 13 is the “true group.” In addition, convergence graphs provide information regarding the time point in the disease course where patients can be reliably classified to their “true group.” The time point in which the two trajectories appear to be horizontal reflects the time point when the group assignments “converge” to their “true groups.” For H&Y stage for both the DodoNA and LONG-PD cohorts, year 9 represents the time point in which group assignment more closely reflects the “true group” assignment (Figures 8B, 9B). For the MMSE score in the DodoNA cohort, this time point is delayed at year 10 (Figure 8A), whereas in the LONG-PD cohort, it occurs earlier in year 8 (Figure 9A). For UPDRS-III and II, that time point is later (Figures 8C,D, 9C,D). Taken together, these results point to the H&Y stage and the MMSE as reliable predictors of trajectory group assignment and identify a point relatively early in the disease trajectory in which group assignment can be made.

**Figure 10:**
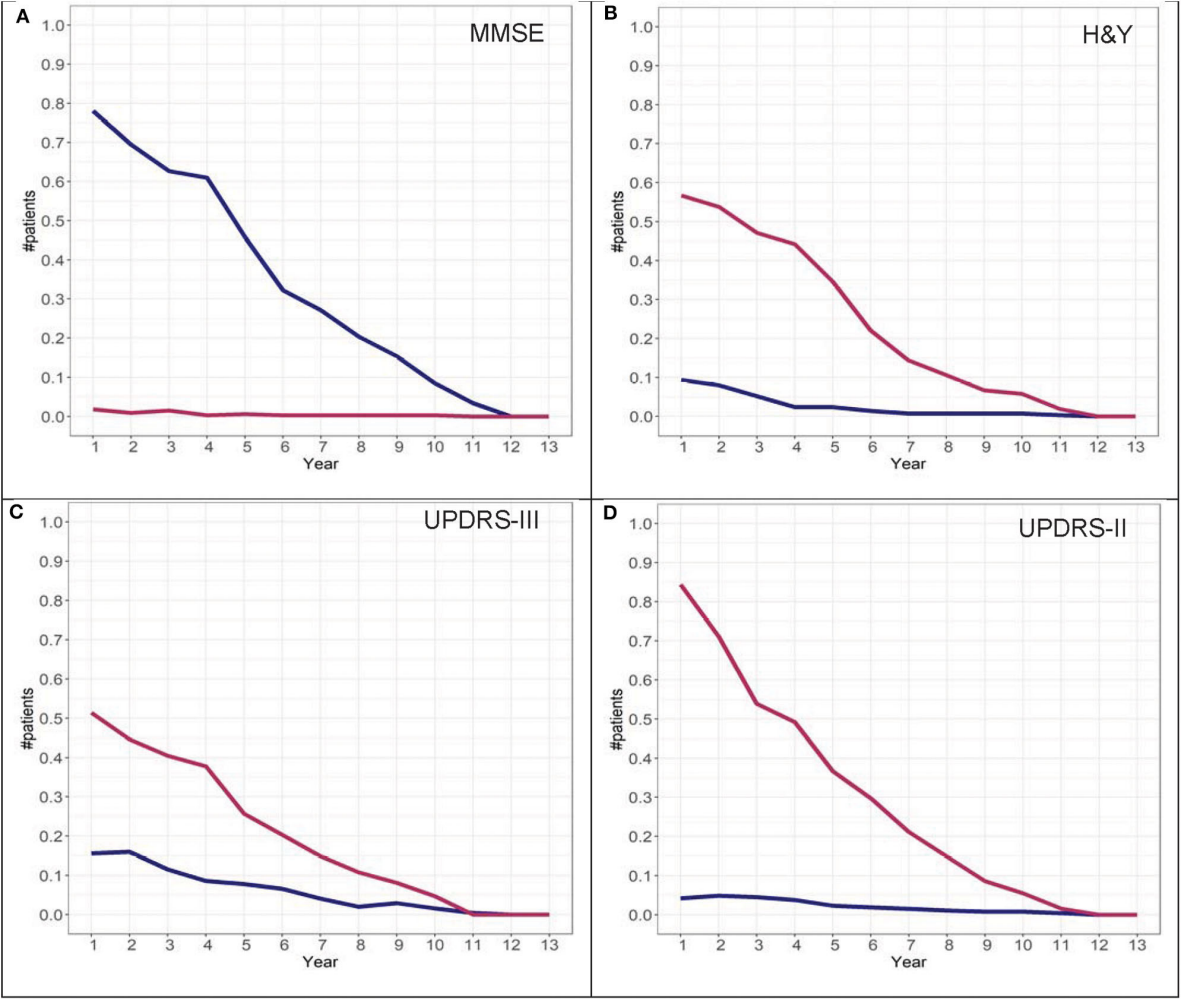
Misclassification scores for MMSE **(A)**, H&Y stage **(B)**, UPDRS-III **(C)**, and UPDRS-II **(D)** for the DodoNA cohort.

**Figure 11:**
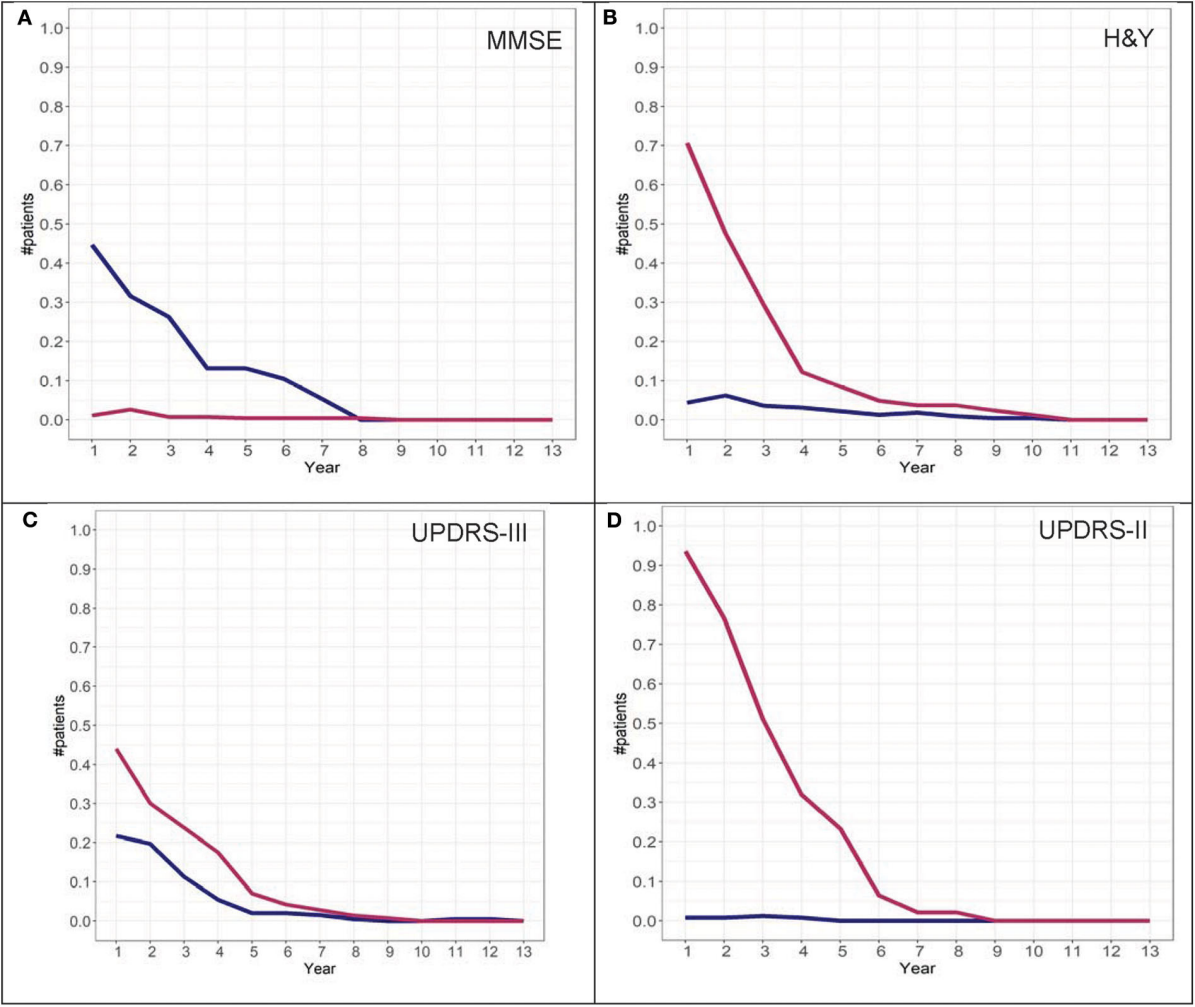
Misclassification scores for MMSE **(A)**, H&Y stage **(B)**, UPDRS-III **(C)**, and UPDRS-II **(D)** for the LONG-PD cohort.

To further investigate the possibility of the misclassification rate for group assignments, misclassification graphs were generated assuming that the assignment at year 13 is the “true group,” complementing the convergence analysis. In the case of H&Y trajectory, convergence was at year 9. At year 9, the misclassification (i.e., 1-accuracy) is 0.05, representing a 5% error rate for group 2 and almost 0% error for group 1 assignment. Based on these graphs, the H&Y stage provides an “acceptable error rate” in both cohorts (Figures 10, 11).

To ascertain the reliability of the analyses, the LONG-PD cohort was used as a training set and the DodoNA cohort as the test set. Both types of analyses provided similar results (data not shown).

#### Covariates Contributing to Trajectory Group Assignment

From the covariates entered into the model, the following contribute to group assignment: older AAO for both cohorts and male sex only for the DodoNA cohort assign patients to the more severe group (group 2) and tremor-predominant disease subtype to the benign group (group 1). Interestingly for tremor scores in the DodoNA cohort only, years of education assigns patients to group 1. Bradykinesia and AAO in the DodoNA cohort only assign patients to group 2. The tremor-predominant subtype in the LONG-PD, but not the DodoNA cohort, assigns patients to group 1. Interestingly, levodopa and dopaminergic therapy are not significant for the DodoNA cohort but are significant for the LONG-PD cohort. Complications of therapy do not contribute to group assignment (data not shown). Cognitive impairment at disease onset likely assigns patients to group 2. The differences noted between the two cohorts may reflect different sample sizes or genetic background effects. Group counts are shown in Table 1 and the effect of covariates in Tables 2, 3. To assess whether the presence of autonomic symptoms contributes to a more rapid and severe course in PD, we also assessed both cohorts for the presence of autonomic symptoms. Orthostatism, urinary incontinence, and constipation were the most prevalent autonomic symptoms. Therefore, we included these in the analysis, individually and in combination. They did not contribute, either individually or in combination, to a more severe disease course in our cohorts.

**Table 1:** Group counts from the DNA Predictions to Improve Neurological Health (DodoNA) and Longitudinal Clinical and Genetic Study of Parkinson's Disease (LONG-PD) cohorts.

**DodoNA cohort**	**Group 1**	**Group 2**
Hoehn and Yahr (H&Y) stage	291 (73.48%)	105 (26.52%)
Mini Mental State Examination (MMSE)	59 (14.9%)	337 (85.1%)
Unified Parkinson's Disease Rating Scale (UPDRS)-III	251 (63.38%)	145 (36.62%)
UPDRS-II	266 (67.17%)	130 (32.83%)
Tremor sub-score	188 (47.47%)	208 (52.53%)
Bradykinesia sub-score	247 (63.01%)	145 (36.99%)
**LONG-PD cohort**	**Group 1**	**Group 2**
H&Y	269 (77.75%)	77 (22.25%)
MMSE	35 (10.12%)	311 (89.88%)
UPDRS-III	203 (58.67%)	143 (41.33%)
UPDRS-II	279 (80.64%)	67 (19.36%)
Tremor sub-score	182 (52.6%)	164 (47.4%)
Bradykinesia sub-score	86 (24.86%)	260 (75.14%)

**Table 2:** Summary statistics, DodoNA cohort: AAO and YOE are continuous covariates and their group values represent within-group means.

**H&Y stage**	**Group 1**	**Group 2**	***P*-value**
AAO^a^	66.0	75.0	<0.0001
YOE^b^	16.0	16.0	<0.0001
FH>1^c^	12	5	0.7817
FH	69	22	0.6594
TDS	71	4	<0.0001
LD^d^	254	90	0.8103
DP^e^	94	23	0.0605
Male sex	221	64	0.0050
**MMSE**			
AAO	75.0	67.0	<0.0001
YOE	15.0	16.0	<0.0001
FH>1	2	15	1.0000
FH	11	80	0.4899
TPS^f^	8	67	0.3354
LD	52	292	0.9176
DP	9	108	0.0141
Male sex	48	237	0.1134
**UPDRS-III (Motor)**			
AAO	67.0	72.0	<0.0001
YOE	16.0	16.0	<0.0001
FH>1	11	6	1.0000
FH	59	32	0.8388
TPS	60	15	0.0014
LD	212	132	0.0871
DP	80	37	0.2221
Male sex	171	114	0.0337
**UPDRS-II (ADL)**			
AAO	68.0	70.0	<0.0001
YOE	16.0	16.0	<0.0001
FH>1	12	5	0.9660
FH	63	28	0.7268
TPS	59	16	0.0266
LD	231	113	1.0000
DP	83	34	0.3592
Male sex	188	97	0.4837
**UPDRS-III Tremor sub-score**			
AAO	69.0000	68.0000	<0.0001
YOE	16.0000	16.0000	<0.0001
FH>1	8	9	1.0000
FH	51	40	0.0809
TDS	0	75	<0.0001
LD	171	173	0.0322
DP	60	57	0.3831
Male sex	130	155	0.2819
**UPDRS-III bradykinesia sub-score**			
AAO	65.0	74.0	<0.0001
YOE	16.0	16.0	<0.0001
FH>1	9	8	0.5569
FH	60	31	0.5354
TPS	67	8	<0.0001
LD	211	133	0.2263
DP	81	36	0.0999
Male sex	177	108	0.8198

*Other covariates are binary, and as such, their values represent within-group sums of positive membership. P-values display statistical significance across groups*.

a *AAO, age at onset*;

b *YOE, years of education*;

c *FH, family history*;

d *LD, levodopa therapy*;

e *DP, dopaminergic therapy*;

f *TPS, tremor-predominant subtype*.

**Table 3:** Summary statistics from the LONG-PD cohort: AAO and YOE are continuous variables and their group values represent within-group means.

	**Group 1**	**Group 2**	***P*-value**
**H&Y stage**			
AAO	61.0	67.0	<0.0001
YOE	12.0	10.0	<0.0001
FH>1	26	6	0.7816
FH	29	11	0.5183
TPS	75	4	<0.0001
LD	248	61	0.0024
DP	142	39	0.8400
Male sex	139	36	0.5273
**MMSE**			
AAO	69.0	62.0	<0.0001
YOE	6.0	12.0	<0.0001
FH >1	1	31	0.2269
FH	2	38	0.4014
TPS	8	71	1.0000
LD	24	285	0.0003
DP	13	168	0.0860
Male sex	16	159	0.6681
**UPDRS-III (Motor)**			
AAO	59.0	67.0	<0.0001
YOE	12.0	10.0	<0.0001
FH>1	18	14	0.9176
FH	23	17	1.0000
TPS	56	23	0.0173
LD	186	123	0.1371
DP	115	66	0.0694
Male sex	105	70	1.0000
**UPDRS-II (ADL)**			
AAO	61.0	67.0	<0.0001
YOE	11.0	10.0	<0.0001
FH>1	26	6	1.0000
FH	32	8	1.0000
TPS	67	12	0.3645
LD	264	45	<0.0001
DP	150	31	0.3337
Male sex	135	40	0.1267
**UPDRS-III tremor sub-score**			
AAO	62.0	63.0	1.0000
YOE	12.0	11.0	1.0000
FH>1	21	11	0.1729
FH	26	14	0.1332
TPS	39	40	0.5981
LD	152	157	0.0005
DP	80	101	0.0015
Male sex	101	74	0.0689
**UPDRS-III bradykinesia sub-score**			
AAO	62.0000	62.0000	1.0000
YOE	10.0000	12.0000	<0.0001
FH>1	9	23	0.8146
FH	12	28	0.5445
TPS	31	48	0.0013
LD	60	249	<0.0001
DP	30	151	0.0003
Male sex	46	129	0.6183

*Other variables are binary, and as such, their values represent within-group sums of positive membership. P-values display statistical significance across groups*.

Genotypes were assessed for the presence of *LRRK2* and *GBA* mutations. The prevalence of *LRRK2* and *GBA* pathogenic variants was 1.89%and 2.96%, respectively. The vast majority of these were in the DodoNA, a United States-based cohort. In combination with the lack of significant contribution of family history to the disease trajectory, this suggests that in these two cohorts, at least some genetic factors do not contribute to the disease trajectory.

## Discussion

Longitudinal monitoring of PD over long time intervals is essential in order to obtain a more accurate characterization of patterns in the disease course and clinical outcomes, as well as to gain insights into disease etiology. Here, we present a group-based trajectory modeling analysis of five ethnically different PD patient cohorts from the United States (the DodoNA cohort) and from Norway, South Korea, Greece, and Sweden (the LONG-PD cohort) within the GEoPD consortium (https://geopd.lcsb.uni.lu/). The trajectory analysis is based on standardized clinical assessment that takes place at annual intervals in the routine office setting. The choice of clinical assessment parameters reflects a consensus among clinicians with different backgrounds and practice modes and which would facilitate data collection and entry using a web-based format. The analysis of a maximum of 13-year disease course identifies two distinct groups: a slower and more benign course and a faster, more malignant course. Clinical predictors of group assignment include male sex, age at disease onset, presence of tremor as a predominant clinical feature, years of education, and cognitive impairment at onset. Interestingly, levodopa/dopaminergic therapy and family history do not contribute to group assignment. The significance of beneficial effect of years of education for assignment to a particular disease trajectory is supported by the findings of Lee et al., which implicate a passive reserve hypothesis for motor/non-motor symptoms of PD (19). The somewhat unexpected lack of contribution of family history in group assignment may reflect the diverse genetic background of the two cohorts.

Adherence to a particular group occurs at mid-stage disease and remains stable thereafter for the study interval. Interestingly, complications of therapy do not appear to contribute to the assignment to individual trajectories. It is interesting to point out that while there is significant overlap between cohorts for the different covariates, there are covariates in which the two cohorts diverge. This may be explained by the different cohort sizes, but it may also reflect different genetic, environmental, and cultural factors. The prevalence of *LRRK2* disease causing variants in sporadic PD has been reported between 0.5 and 2% (20, 21) and that of *GBA* between 2.3 and 9.4% (22) in the U.S. population, similar to what we find in our cohort. It seems unlikely that the low percentage of *LRRK2* and *GBA* disease-causing variants drives trajectory classification as there is a lack of contribution of family history to trajectory classification. This suggests that genetic factors are not likely to have at best a modest effect.

The GBTM analysis presented here has several strengths: (a) it employs easily assessed standardized clinical parameters that can be assessed at annual intervals and identifies predictable patterns of disease progression; (b) the analysis is performed over a long disease duration (maximum of 13 years); (c) it identifies individual clinical predictors of trajectory patterns; (d) the accurate clinical phenotypic characterization provides an essential background for genotype–phenotype correlations, currently ongoing in our study; (e) it provides an informative template for large-scale clinical and genomic studies.

Our study has also some limitations. Since the intent of this study was to assess measures that could be evaluated in a routine clinical setting, a limitation is its assessment of a narrower spectrum of phenotypic characteristics than other comprehensively studied cohorts such as the PPMI, DeNOPA, and LABS-PD cohorts (2–6, 23). Specifically, in our cohorts, CSF analyses, SPECT scans, quantitative olfactory assessment, and polysomnograms were not obtained routinely. Since the study protocols of other longitudinally studied cohorts vary in aims and scope, direct comparisons with our study are challenging. These issues would be better addressed by a meta-analysis.

A second limitation of our study is the lack of autopsy data. However, over a quarter of participants underwent SPECT scans that were abnormal. In the absence of autopsy data, an abnormal SPECT scan in the context of clinically definite PD (Bower criteria) confirms the clinical diagnosis. In that context, it should also be pointed out that the diagnosis of PD in our study was assessed and confirmed at each annual interval.

A strength of this study is that detailed information on comorbidities, head injury, complications of dopaminergic therapy, autonomic dysfunction (orthostatic symptoms, anhidrosis/hyperhidrosis, urinary incontinence), sleep disorders, dysphagia, anxiety, and depression have been, and continue to be, collected at annual intervals. As the study is ongoing, these will continue to be analyzed to inform conclusions regarding the spectrum of factors that contribute to the disease course in intervals longer than 5 years. It is important to point out that the focus of the analysis presented here is to identify individual, clinical parameters that reflect the cardinal features of the disease as well as assess the effect of other covariates on those parameters. Furthermore, it is important to stress that the clinical data collected in the DodoNA and LONG-PD cohorts are pragmatic and can be easily collected within routine clinical practice settings worldwide. Identifying what features in this simplified, reproducible set of clinical parameters can predict disease course complements findings from other longitudinally followed disease cohorts.

In conclusion, the longitudinal study of the DodoNA and the LONG-PD cohorts combines clinically meaningful, easily obtainable information from ethnically different PD cohorts and demonstrates that clinical parameters assessed in the routine office setting can help predict clinical outcomes in PD as well as inform our understanding of the underlying neurodegenerative process. Large international consortia to understand genetic risk factors contributing to PD have been formed where phenotypic information is sketchy and often minimal. This work demonstrates that a detailed phenotypic characterization is essential in informing and interpreting the data from such consortia. The development of the LONG-PD protocol has led to the adoption of a somewhat simplified version of phenotypic information collection by a majority of the GEoPD participating sites and can be easily adapted for genomic information obtained by other international consortia. Ongoing genotype–phenotype analyses will identify molecular predictors of the disease trajectories. Longer longitudinal follow-up of >10 years will help determine whether the adherence to the identified trajectories remains stable or whether splintering occurs as the disease process advances.

## Data Availability Statement

The datasets generated for this study are available on request to the corresponding author.

## Ethics Statement

The studies involving human participants were reviewed and approved by Ethical Committee for Central Norway, Ethics committee of the University Hospital of Larissa, Regional Ethics Review Board in Stockholm, Sweden, Institutional Review Board Of Asan Medical Center, Institutional Review Board NorthShore University HealthSystem. The patients/participants provided their written informed consent to participate in this study.

## Author Contributions

KM, RF, and DM contributed in the conception and design of the study. KM wrote the first draft of the manuscript. KS wrote sections of the manuscript. KM, JA, SC, ED, KW, APP, BS, DM, LG, AP, and NK collected the data. BH and AE built the EMR system. ST and GW performed the statistical analysis. JW performed the genomic analysis. All authors contributed to the manuscript revision, read, and approved the submitted version.

## Conflict of Interest

The authors declare that the research was conducted in the absence of any commercial or financial relationships that could be construed as a potential conflict of interest.

## Supplementary Material

Supplemental Information: LONG-PD Study Protocol.
