# Supplementary material for: Longitudinal Monitoring of Parkinson's Disease in Different Ethnic Cohorts: The DodoNA and LONG-PD Study
Source: Front Neurol. 2020 Jul 7;11:548. doi: 10.3389/fneur.2020.00548 (PMC7358533; doi:10.3389/fneur.2020.00548)
Supplement: Supplementary file 1 [file Data_Sheet_1.PDF]

Demographics

Record ID ______________________________

Site PI
◯
◯
◯
◯
◯
◯
◯
◯
◯
◯
◯
◯
◯
◯
◯
◯
◯
◯
◯
◯

Study ID ______________________________
(study specific; e.g. EH#)

Year of Birth

- ◯ 1914
- ◯ 1915
- ◯ 1916
- ◯ 1917
- ◯ 1918
- ◯ 1919
- ◯ 1920
- ◯ 1921
- ◯ 1922
- ◯ 1923
- ◯ 1924
- ◯ 1925
- ◯ 1926
- ◯ 1927
- ◯ 1928
- ◯ 1929
- ◯ 1930
- ◯ 1931
- ◯ 1932
- ◯ 1933
- ◯ 1934
- ◯ 1935
- ◯ 1936
- ◯ 1937
- ◯ 1938
- ◯ 1939
- ◯ 1940
- ◯ 1941
- ◯ 1942
- ◯ 1943
- ◯ 1944
- ◯ 1945
- ◯ 1946
- ◯ 1947
- ◯ 1948
- ◯ 1949
- ◯ 1950
- ◯ 1951
- ◯ 1952
- ◯ 1953
- ◯ 1954
- ◯ 1955
- ◯ 1956
- ◯ 1957
- ◯ 1958
- ◯ 1959
- ◯ 1960
- ◯ 1961
- ◯ 1962
- ◯ 1963
- ◯ 1964
- ◯ 1965
- ◯ 1966
- ◯ 1967
- ◯ 1968
- ◯ 1969
- ◯ 1970
- ◯ 1971
- ◯ 1972
- ◯ 1973
- ◯ 1974
- ◯ 1975
- ◯ 1976
- ◯ 1977
- ◯ 1978
- ◯ 1979
- ◯ 1980
- ◯ 1981
- ◯ 1982
- ◯ 1983

	◯ 1984<br>◯ 1985<br>◯ 1986<br>◯ 1987<br>◯ 1988<br>◯ 1989<br>◯ 1990<br>◯ 1991<br>◯ 1992<br>◯ 1993<br>◯ 1994
Gender	◯ M<br>◯ F
Race	◯ American Indian or Alaska Native<br>◯ African American<br>◯ Asian<br>◯ Caucasian<br>◯ Pacific Islander/Hawaiian Native<br>◯ Other<br>◯ Unknown
Ethnicity	◯ Hispanic<br>◯ Non-Hispanic<br>◯ Unknown

Other

Examiner Last Name __________________________________

Examiner First Name __________________________________

Year of visit
- ◯ 2015
- ◯ 2016
- ◯ 2017
- ◯ 2018
- ◯ 2019
- ◯ 2020
- ◯ 2021
- ◯ 2022
- ◯ 2023
- ◯ 2024
- ◯ 2025
- ◯ 2026
- ◯ 2027
- ◯ 2028
- ◯ 2029
- ◯ 2030

Age at visit __________________________________

Vital signs

Systolic blood pressure mm Hg (sitting) __________________________________

Diastolic blood pressure mm Hg (sitting) __________________________________

Pulse per minute (sitting) __________________________________

Weight (kg) __________________________________

Height (cm) __________________________________

BMI __________________________________

Current Medications

Are you currently taking any medications for Parkinson's Disease?
- ◯ Yes
- ◯ No

Please specify:

- ☐ Amantadine Hydrochloride 100 MG Oral Capsule
- ☐ Amantadine Hydrochloride 100 MG Oral Tablet
- ☐ Amantadine Hydrochloride 10 MG/ML Oral Solution
- ☐ Apomorphine 10 MG/ML Injectable Solution
- ☐ Apomorphine 2 MG Sublingual Tablet
- ☐ Apomorphine 3 MG Sublingual Tablet
- ☐ Apomorphine 6 MG Disintegrating Tablet
- ☐ 3 ML Apomorphine 10 MG/ML Prefilled Syringe
- ☐ 10 ML Apomorphine 5 MG/ML Prefilled Syringe
- ☐ Benserazide 12.5 MG / Levodopa 50 MG Oral Capsule
- ☐ Benserazide 12.5 MG / Levodopa 50 MG Oral Tablet
- ☐ Benserazide 25 MG / Levodopa 100 MG Extended Release Capsule
- ☐ Benserazide 25 MG / Levodopa 100 MG Oral Capsule
- ☐ Benserazide 25 MG / Levodopa 100 MG Oral Tablet
- ☐ Benserazide 50 MG / Levodopa 200 MG Oral Capsule
- ☐ Benserazide 50 MG / Levodopa 200 MG Oral Tablet
- ☐ Benztropine Mesylate 0.5 MG Oral Tablet
- ☐ Benztropine Mesylate 1 MG Oral Tablet
- ☐ Benztropine Mesylate 1 MG/ML Injectable Solution
- ☐ Benztropine Mesylate 2 MG Oral Tablet
- ☐ Carbidopa 25 MG Oral Tablet
- ☐ Carbidopa 10 MG / Levodopa 100 MG Disintegrating Tablet
- ☐ Carbidopa 10 MG / Levodopa 100 MG Oral Tablet
- ☐ Carbidopa 12.5 MG / Levodopa 50 MG Oral Tablet
- ☐ Carbidopa 25 MG / Levodopa 100 MG Disintegrating Tablet
- ☐ Carbidopa 25 MG / Levodopa 100 MG Extended Release Tablet
- ☐ Carbidopa 25 MG / Levodopa 100 MG Oral Tablet
- ☐ Carbidopa 25 MG / Levodopa 250 MG Disintegrating Tablet
- ☐ Carbidopa 25 MG / Levodopa 250 MG Oral Tablet
- ☐ Carbidopa 50 MG / Levodopa 200 MG Extended Release Tablet
- ☐ Carbidopa/Levodopa 23.75/95 Extended Release Capsules
- ☐ Carbidopa/Levodopa 36.25/145 Extended Release Capsules
- ☐ Carbidopa/Levodopa 48.75/195 Extended Release Capsules
- ☐ Carbidopa/Levodopa 61.25/245 Extended Release Capsules
- ☐ Carbidopa/Levodopa 4.63/20 mg per mL enteral suspension
- ☐ Carbidopa 12.5 MG / Entacapone 200 MG / Levodopa 50 MG Oral Tablet
- ☐ Carbidopa 18.75 MG / Entacapone 200 MG / Levodopa 75 MG Oral Tablet
- ☐ Carbidopa 25 MG / Entacapone 200 MG / Levodopa 100 MG Oral Tablet
- ☐ Carbidopa 31.25 MG / Entacapone 200 MG / Levodopa 125 MG Oral Tablet
- ☐ Carbidopa 37.5 MG / Entacapone 200 MG / Levodopa 150 MG Oral Tablet
- ☐ Carbidopa 50 MG / Entacapone 200 MG / Levodopa 200 MG Oral Tablet
- ☐ Clozapine 12.5 MG Disintegrating Tablet
- ☐ Clozapine 12.5 MG Oral Tablet
- ☐ Clozapine 25 MG Disintegrating Tablet
- ☐ Clozapine 25 MG Oral Tablet
- ☐ Clozapine 50 MG Disintegrating Tablet
- ☐ Clozapine 50 MG Oral Tablet
- ☐ Clozapine 50 MG/ML Oral Suspension
- ☐ Clozapine 100 MG Disintegrating Tablet
- ☐ Clozapine 100 MG Oral Tablet
- ☐ Clozapine 150 MG Disintegrating Tablet
- ☐ Clozapine 200 MG Disintegrating Tablet
- ☐ Clozapine 200 MG Oral Tablet
- ☐ Entacapone 200 MG Oral Tablet
- ☐ Pramipexole Dihydrochloride 0.125 MG Oral Tablet
- ☐ Pramipexole Dihydrochloride 0.25 MG Oral Tablet
- ☐ Pramipexole Dihydrochloride 0.5 MG Oral Tablet
- ☐ Pramipexole Dihydrochloride 0.75 MG Oral Tablet
- ☐ Pramipexole Dihydrochloride 1 MG Oral Tablet
- ☐ Pramipexole Dihydrochloride 1.5 MG Oral Tablet
- ☐ 24 HR Pramipexole Dihydrochloride 0.26 MG Extended Release Tablet
- ☐ 24 HR Pramipexole Dihydrochloride 0.375 MG Extended Release Tablet
- ☐ 24 HR Pramipexole Dihydrochloride 0.52 MG Extended Release Tablet
- ☐ 24 HR Pramipexole Dihydrochloride 0.75 MG Extended Release Tablet
- ☐ 24 HR Pramipexole Dihydrochloride 1.05 MG Extended Release Tablet
- ☐ 24 HR Pramipexole Dihydrochloride 1.5 MG Extended Release Tablet
- ☐ 24 HR Pramipexole Dihydrochloride 1.57 MG Extended Release Tablet
- ☐ 24 HR Pramipexole Dihydrochloride 2.1 MG Extended Release Tablet

☐ 24 HR Pramipexole Dihydrochloride 2.25 MG Extended Release Tablet
☐ 24 HR Pramipexole Dihydrochloride 2.62 MG Extended Release Tablet
☐ 24 HR Pramipexole Dihydrochloride 3 MG Extended Release Tablet
☐ 24 HR Pramipexole Dihydrochloride 3.15 MG Extended Release Tablet
☐ 24 HR Pramipexole Dihydrochloride 3.75 MG Extended Release Tablet
☐ 24 HR Pramipexole Dihydrochloride 4.5 MG Extended Release Tablet
☐ Quetiapine 25 MG Oral Tablet
☐ Quetiapine 50 MG Oral Tablet
☐ Quetiapine 100 MG Oral Tablet
☐ Quetiapine 150 MG Oral Tablet
☐ Quetiapine 200 MG Oral Tablet
☐ Quetiapine 300 MG Oral Tablet
☐ Quetiapine 400 MG Oral Tablet
☐ 24 HR Quetiapine 50 MG Extended Release Tablet
☐ 24 HR Quetiapine 150 MG Extended Release Tablet
☐ 24 HR Quetiapine 200 MG Extended Release Tablet
☐ 24 HR Quetiapine 300 MG Extended Release Tablet
☐ 24 HR Quetiapine 400 MG Extended Release Tablet
☐ Rasagiline 0.5 MG Oral Tablet
☐ Rasagiline 1 MG Oral Tablet
☐ Ropinirole 0.25 MG Oral Tablet
☐ Ropinirole 0.5 MG Oral Tablet
☐ Ropinirole 1 MG Oral Tablet
☐ Ropinirole 2 MG Oral Tablet
☐ Ropinirole 3 MG Oral Tablet
☐ Ropinirole 4 MG Oral Tablet
☐ Ropinirole 5 MG Oral Tablet
☐ 24 HR Ropinirole 2 MG Extended Release Tablet
☐ 24 HR Ropinirole 3 MG Extended Release Tablet
☐ 24 HR Ropinirole 4 MG Extended Release Tablet
☐ 24 HR Ropinirole 6 MG Extended Release Tablet
☐ 24 HR Ropinirole 8 MG Extended Release Tablet
☐ 24 HR Ropinirole 12 MG Extended Release Tablet
☐ {2 (Ropinirole 0.25 MG Oral Tablet) / 5 (Ropinirole 0.5 MG Oral Tablet) / 7 (Ropinirole 1 MG Oral Tablet) } Pack
☐ 24 HR Rotigotine 1MG/24HRS (0.0417 MG/HR) Transdermal Patch
☐ 24 HR Rotigotine 2MG/24HRS (0.0833 MG/HR) Transdermal Patch
☐ 24 HR Rotigotine 3MG/24HRS (0.125 MG/HR) Transdermal Patch
☐ 24 HR Rotigotine 4MG/24HRS (0.167 MG/HR) Transdermal Patch
☐ 24 HR Rotigotine 6MG/24HRS (0.25 MG/HR) Transdermal Patch
☐ 24 HR Rotigotine 8MG/24HRS (0.333 MG/HR) Transdermal Patch
☐ {7 (24 HR Rotigotine 0.0833 MG/HR Transdermal Patch) / 7 (24 HR Rotigotine 0.167 MG/HR Transdermal Patch) } Pack
☐ Selegiline Hydrochloride 1.25 MG Disintegrating Tablet
☐ Selegiline Hydrochloride 1.25 MG Oral Tablet
☐ Selegiline Hydrochloride 5 MG Oral Tablet
☐ Selegiline Hydrochloride 10 MG Oral Tablet
☐ Selegiline Hydrochloride 2 MG/ML Oral Solution
☐ Selegiline Hydrochloride 5 MG Oral Capsule
☐ 24 HR Selegiline Hydrochloride 0.25 MG/HR Transdermal Patch
☐ 24 HR Selegiline Hydrochloride 0.375 MG/HR Transdermal Patch
☐ 24 HR Selegiline Hydrochloride 0.5 MG/HR Transdermal Patch
☐ Tolcapone 100 MG Oral Tablet
☐ Tolcapone 200 MG Oral Tablet
☐ Trihexyphenidyl Hydrochloride 2 MG Oral Tablet
☐ Trihexyphenidyl Hydrochloride 5 MG Oral Tablet
☐ Trihexyphenidyl Hydrochloride 5 MG Extended Release Capsule
☐ Trihexyphenidyl Hydrochloride 0.4 MG/ML Oral Solution
☐ Trihexyphenidyl Hydrochloride 1 MG/ML Oral Solution

Please specify the number of unit doses total per day for Apomorphine 10 MG/ML Injectable Solution ______________________________

Please specify the number of unit doses total per day for Apomorphine 2 MG Sublingual Tablet ______________________________

Please specify the number of unit doses total per day for Apomorphine 3 MG Sublingual Tablet ______________________________

Please specify the number of unit doses total per day for 3 ML Apomorphine 10 MG/ML Prefilled Syringe ________________________________

Please specify the number of unit doses total per day for 10 ML Apomorphine 5 MG/ML Prefilled Syringe ________________________________

Please specify the number of unit doses total per day for Apomorphine 6 MG Disintegrating Tablet ________________________________

Please specify the number of unit doses total per day for Carbidopa 10 MG / Levodopa 100 MG Disintegrating Tablet ________________________________

Please specify the number of unit doses total per day for Carbidopa 10 MG / Levodopa 100 MG Oral Tablet ________________________________

Please specify the number of unit doses total per day for Carbidopa 12.5 MG / Entacapone 200 MG / Levodopa 50 MG Oral Tablet ________________________________

Please specify the number of unit doses total per day for Carbidopa 12.5 MG / Levodopa 50 MG Oral Tablet ________________________________

Please specify the number of unit doses total per day for Carbidopa 18.75 MG / Entacapone 200 MG / Levodopa 75 MG Oral Tablet ________________________________

Please specify the number of unit doses total per day for Carbidopa 25 MG / Entacapone 200 MG / Levodopa 100 MG Oral Tablet ________________________________

Please specify the number of unit doses total per day for Carbidopa 25 MG / Levodopa 100 MG Disintegrating Tablet ________________________________

Please specify the number of unit doses total per day for Carbidopa 25 MG / Levodopa 100 MG Extended Release Tablet ________________________________

Please specify the number of unit doses total per day for Carbidopa 25 MG / Levodopa 100 MG Oral Tablet ________________________________

Please specify the number of unit doses total per day for Carbidopa 25 MG / Levodopa 250 MG Disintegrating Tablet ________________________________

Please specify the number of unit doses total per day for Carbidopa 25 MG / Levodopa 250 MG Oral Tablet ________________________________

Please specify the number of unit doses total per day for Carbidopa 25 MG Oral Tablet ________________________________

Please specify the number of unit doses total per day for Carbidopa 31.25 MG / Entacapone 200 MG / Levodopa 125 MG Oral Tablet ________________________________

Please specify the number of unit doses total per day for Carbidopa 37.5 MG / Entacapone 200 MG / Levodopa 150 MG Oral Tablet ________________________________

Please specify the number of unit doses total per day for Carbidopa 50 MG / Entacapone 200 MG / Levodopa 200 MG Oral Tablet ________________________________

Please specify the number of unit doses total per day for Carbidopa 50 MG / Levodopa 200 MG Extended Release Tablet ________________________________

Please specify the number of unit doses total per day for Benserazide 12.5 MG / Levodopa 50 MG Oral Capsule ______________________

Please specify the number of unit doses total per day for Benserazide 12.5 MG / Levodopa 50 MG Oral Tablet ______________________

Please specify the number of unit doses total per day for Benserazide 25 MG / Levodopa 100 MG Extended Release Capsule ______________________

Please specify the number of unit doses total per day for Benserazide 25 MG / Levodopa 100 MG Oral Capsule ______________________

Please specify the number of unit doses total per day for Benserazide 25 MG / Levodopa 100 MG Oral Tablet ______________________

Please specify the number of unit doses total per day for Benserazide 50 MG / Levodopa 200 MG Oral Capsule ______________________

Please specify the number of unit doses total per day for Benserazide 50 MG / Levodopa 200 MG Oral Tablet ______________________

Please specify the number of unit doses total per day for Entacapone 200 MG Oral Tablet ______________________

Please specify the number of unit doses total per day for Pramipexole dihydrochloride 0.125 MG Oral Tablet ______________________

Please specify the number of unit doses total per day for Pramipexole dihydrochloride 0.25 MG Oral Tablet ______________________

Please specify the number of unit doses total per day for 24 HR Pramipexole Dihydrochloride 0.26 MG Extended Release Tablet ______________________

Please specify the number of unit doses total per day for 24 HR Pramipexole Dihydrochloride 0.375 MG Extended Release Tablet ______________________

Please specify the number of unit doses total per day for 24 HR Pramipexole Dihydrochloride 0.52 MG Extended Release Tablet ______________________

Please specify the number of unit doses total per day for Pramipexole Dihydrochloride 0.5 MG Oral Tablet ______________________

Please specify the number of unit doses total per day for Pramipexole Dihydrochloride 0.75 MG Oral Tablet ______________________

Please specify the number of unit doses total per day for 24 HR Pramipexole Dihydrochloride 0.75 MG Extended Release Tablet ______________________

Please specify the number of unit doses total per day for 24 HR Pramipexole Dihydrochloride 1.05 MG Extended Release Tablet ______________________

Please specify the number of unit doses total per day for Pramipexole Dihydrochloride 1 MG Oral Tablet ______________________

Please specify the number of unit doses total per day for Pramipexole Dihydrochloride 1.5 MG Oral Tablet ______________________

Please specify the number of unit doses total per day for 24 HR Pramipexole Dihydrochloride 1.5 MG Extended Release Tablet ______________________

Please specify the number of unit doses total per day for 24 HR Pramipexole Dihydrochloride 1.57 MG Extended Release Tablet ________________

Please specify the number of unit doses total per day for 24 HR Pramipexole Dihydrochloride 2.1 MG Extended Release Tablet ________________

Please specify the number of unit doses total per day for 24 HR Pramipexole Dihydrochloride 2.25 MG Extended Release Tablet ________________

Please specify the number of unit doses total per day for 24 HR Pramipexole Dihydrochloride 2.62 MG Extended Release Tablet ________________

Please specify the number of unit doses total per day for 24 HR Pramipexole Dihydrochloride 3 MG Extended Release Tablet ________________

Please specify the number of unit doses total per day for 24 HR Pramipexole Dihydrochloride 3.15 MG Extended Release Tablet ________________

Please specify the number of unit doses total per day for 24 HR Pramipexole Dihydrochloride 3.75 MG Extended Release Tablet ________________

Please specify the number of unit doses total per day for 24 HR Pramipexole Dihydrochloride 4.5 MG Extended Release Tablet ________________

Please specify the number of unit doses total per day for Rasagiline 0.5 MG Oral Tablet ________________

Please specify the number of unit doses total per day for Rasagiline 1 MG Oral Tablet ________________

Please specify the number of unit doses total per day for Ropinirole 0.5 MG Oral Tablet ________________

Please specify the number of unit doses total per day for Ropinirole 5 MG Oral Tablet ________________

Please specify the number of unit doses total per day for Ropinirole 0.25 MG Oral Tablet ________________

Please specify the number of unit doses total per day for Ropinirole 1 MG Oral Tablet ________________

Please specify the number of unit doses total per day for 24 HR Ropinirole 12 MG Extended Release Tablet ________________

Please specify the number of unit doses total per day for Ropinirole 2 MG Oral Tablet ________________

Please specify the number of unit doses total per day for 24 HR Ropinirole 2 MG Extended Release Tablet ________________

Please specify the number of unit doses total per day for Ropinirole 3 MG Oral Tablet ________________

Please specify the number of unit doses total per day for 24 HR Ropinirole 3 MG Extended Release Tablet ________________

Please specify the number of unit doses total per day for Ropinirole 4 MG Oral Tablet ________________

Please specify the number of unit doses total per day for 24 HR Ropinirole 4 MG Extended Release Tablet ______________________________

Please specify the number of unit doses total per day for 24 HR Ropinirole 6 MG Extended Release Tablet ______________________________

Please specify the number of unit doses total per day for 24 HR Ropinirole 8 MG Extended Release Tablet ______________________________

Please specify the number of unit doses total per day for {2 (Ropinirole 0.25 MG Oral Tablet) / 5 (Ropinirole 0.5 MG Oral Tablet) / 7 (Ropinirole 1 MG Oral Tablet) } Pack ______________________________

Please specify the number of unit doses total per day for 24 HR Rotigotine 1MG/24HRS (0.0417 MG/HR) Transdermal Patch ______________________________

Please specify the number of unit doses total per day for 24 HR Rotigotine 2MG/24HRS (0.0833 MG/HR) Transdermal Patch ______________________________

Please specify the number of unit doses total per day for 24 HR Rotigotine 3MG/24HRS (0.125 MG/HR) Transdermal Patch ______________________________

Please specify the number of unit doses total per day for 24 HR Rotigotine 4MG/24HRS (0.167 MG/HR) Transdermal Patch ______________________________

Please specify the number of unit doses total per day for 24 HR Rotigotine 6MG/24HRS (0.25 MG/HR) Transdermal Patch ______________________________

Please specify the number of unit doses total per day for 24 HR Rotigotine 8MG/24HRS (0.333 MG/HR) Transdermal Patch ______________________________

Please specify the number of unit doses total per day for {7 (24 HR Rotigotine 0.0833 MG/HR Transdermal Patch) / 7 (24 HR Rotigotine 0.167 MG/HR Transdermal Patch) } Pack ______________________________

Please specify the number of unit doses total per day for 24 HR Selegiline Hydrochloride 0.5 MG/HR Transdermal Patch ______________________________

Please specify the number of unit doses total per day for Selegiline Hydrochloride 1.25 MG Disintegrating Tablet ______________________________

Please specify the number of unit doses total per day for Selegiline Hydrochloride 1.25 MG Oral Tablet ______________________________

Please specify the number of unit doses total per day for Selegiline Hydrochloride 10 MG Oral Tablet ______________________________

Please specify the number of unit doses total per day for Selegiline Hydrochloride 2 MG/ML Oral Solution ______________________________

Please specify the number of unit doses total per day for Selegiline Hydrochloride 5 MG Oral Capsule ______________________________

Please specify the number of unit doses total per day for Selegiline Hydrochloride 5 MG Oral Tablet ______________________________

Please specify the number of unit doses total per day for 24 HR Selegiline Hydrochloride 0.25 MG/HR Transdermal Patch ______________________________

Please specify the number of unit doses total per day for 24 HR Selegiline Hydrochloride 0.375 MG/HR Transdermal Patch ______________________________

Please specify the number of unit doses total per day for Tolcapone 100 MG Oral Tablet ______________________________

Please specify the number of unit doses total per day for Tolcapone 200 MG Oral Tablet ______________________________

Please specify the number of unit doses total per day for Amantadine Hydrochloride 100 MG Oral Capsule ______________________________

Please specify the number of unit doses total per day for Amantadine Hydrochloride 100 MG Oral Tablet ______________________________

Please specify the number of unit doses total per day for Amantadine Hydrochloride 10 MG/ML Oral Solution ______________________________

Please specify the number of unit doses total per day for Benztropine Mesylate 1 MG Oral Tablet ______________________________

Please specify the number of unit doses total per day for Benztropine Mesylate 1 MG/ML Injectable Solution ______________________________

Please specify the number of unit doses total per day for Benztropine Mesylate 2 MG Oral Tablet ______________________________

Please specify the number of unit doses total per day for Benztropine Mesylate 0.5 MG Oral Tablet ______________________________

Please specify the number of unit doses total per day for Clozapine 100 MG Disintegrating Tablet ______________________________

Please specify the number of unit doses total per day for Clozapine 100 MG Oral Tablet ______________________________

Please specify the number of unit doses total per day for Clozapine 12.5 MG Disintegrating Tablet ______________________________

Please specify the number of unit doses total per day for Clozapine 12.5 MG Oral Tablet ______________________________

Please specify the number of unit doses total per day for Clozapine 150 MG Disintegrating Tablet ______________________________

Please specify the number of unit doses total per day for Clozapine 200 MG Disintegrating Tablet ______________________________

Please specify the number of unit doses total per day for Clozapine 200 MG Oral Tablet ______________________________

Please specify the number of unit doses total per day for Clozapine 25 MG Disintegrating Tablet ______________________________

Please specify the number of unit doses total per day for Clozapine 25 MG Oral Tablet ______________________________

Please specify the number of unit doses total per day for Clozapine 50 MG Disintegrating Tablet ______________________________

Please specify the number of unit doses total per day for Clozapine 50 MG Oral Tablet ______________________________

Please specify the number of unit doses total per day for Clozapine 50 MG/ML Oral Suspension ______________________

Please specify the number of unit doses total per day for Quetiapine 100 MG Oral Tablet ______________________

Please specify the number of unit doses total per day for Quetiapine 150 MG Oral Tablet ______________________

Please specify the number of unit doses total per day for Quetiapine 200 MG Oral Tablet ______________________

Please specify the number of unit doses total per day for Quetiapine 25 MG Oral Tablet ______________________

Please specify the number of unit doses total per day for Quetiapine 300 MG Oral Tablet ______________________

Please specify the number of unit doses total per day for Quetiapine 400 MG Oral Tablet ______________________

Please specify the number of unit doses total per day for Quetiapine 50 MG Oral Tablet ______________________

Please specify the number of unit doses total per day for 24 HR Quetiapine 150 MG Extended Release Tablet ______________________

Please specify the number of unit doses total per day for 24 HR Quetiapine 200 MG Extended Release Tablet ______________________

Please specify the number of unit doses total per day for 24 HR Quetiapine 300 MG Extended Release Tablet ______________________

Please specify the number of unit doses total per day for 24 HR Quetiapine 400 MG Extended Release Tablet ______________________

Please specify the number of unit doses total per day for 24 HR Quetiapine 50 MG Extended Release Tablet ______________________

Please specify the number of unit doses total per day for Trihexyphenidyl Hydrochloride 2 MG Oral Tablet ______________________

Please specify the number of unit doses total per day for Trihexyphenidyl Hydrochloride 0.4 MG/ML Oral Solution ______________________

Please specify the number of unit doses total per day for Trihexyphenidyl Hydrochloride 5 MG Extended Release Capsule ______________________

Please specify the number of unit doses total per day for Trihexyphenidyl Hydrochloride 5 MG Oral Tablet ______________________

Please specify the number of unit doses total per day for Trihexyphenidyl Hydrochloride 1 MG/ML Oral Solution ______________________

Other Medications (class)

- ☐ Antihypertensive
- ☐ Vasopressor/fludrocortisone
- ☐ Statin
- ☐ Aspirin
- ☐ NSAID
- ☐ Corticosteroid (other than fludrocortisone)
- ☐ Antidepressant
- ☐ Benzodiazepine
- ☐ Antipsychotic
- ☐ Anti-cholinesterase inhibitor
- ☐ NMDA antagonist
- ☐ None of the Above

Demographic Information

Years of education

- ◯ 0
- ◯ 1
- ◯ 2
- ◯ 3
- ◯ 4
- ◯ 5
- ◯ 6
- ◯ 7
- ◯ 8
- ◯ 9
- ◯ 10
- ◯ 11
- ◯ 12
- ◯ 13
- ◯ 14
- ◯ 15
- ◯ 16
- ◯ 17
- ◯ 18
- ◯ 19
- ◯ 20
- ◯ 21
- ◯ 22
- ◯ 23
- ◯ 24
- ◯ 25
- ◯ 26
- ◯ 27
- ◯ 28
- ◯ 29
- ◯ 30+

Working?

- ◯ Yes
- ◯ No

Current occupation ______________________________

Year stopped working

◯ Don't know
◯ 1960
◯ 1961
◯ 1962
◯ 1963
◯ 1964
◯ 1965
◯ 1966
◯ 1967
◯ 1968
◯ 1969
◯ 1970
◯ 1971
◯ 1972
◯ 1973
◯ 1974
◯ 1975
◯ 1976
◯ 1977
◯ 1978
◯ 1979
◯ 1980
◯ 1981
◯ 1982
◯ 1983
◯ 1984
◯ 1985
◯ 1986
◯ 1987
◯ 1988
◯ 1989
◯ 1990
◯ 1991
◯ 1992
◯ 1993
◯ 1994
◯ 1995
◯ 1996
◯ 1997
◯ 1998
◯ 1999
◯ 2000
◯ 2001
◯ 2002
◯ 2003
◯ 2004
◯ 2005
◯ 2006
◯ 2007
◯ 2008
◯ 2009
◯ 2010
◯ 2011
◯ 2012
◯ 2013
◯ 2014
◯ 2015
◯ 2016
◯ 2017
◯ 2018
◯ 2019
◯ 2020
◯ 2021
◯ 2022
◯ 2023
◯ 2024
◯ 2025
◯ 2026
◯ 2027
◯ 2028

◯ 2029
◯ 2030

Reason stopped working
◯ PD related
◯ not PD related

Last occupation ______________________

Tobacco use
◯ Current
◯ Former
◯ Never

Caffeine use
◯ Current
◯ Former
◯ Never

Marital Status
◯ Divorced
◯ Engaged
◯ Legally Separated
◯ Life Partner
◯ Married
◯ Separated (not legally)
◯ Single
◯ Widowed
◯ Unknown

Exposures

Have you ever used any type of pesticides (herbicides, insecticides, fungicides) on a regular basis (multiple applications in a year)
◯ Yes
◯ No

Present or past
◯ Past
◯ Present

Occupational/Hobby/Both
◯ Occupational
◯ Hobby
◯ Both

History of Head Injury

Have you ever had a head injury where you lost consciousness
◯ Yes
◯ No

List number of head injuries that resulted in loss of consciousness
◯ 1
◯ 2
◯ 3
◯ 4
◯ 5
◯ 5+

Year(s)

☐ 1930 ☐ 1931 ☐ 1932 ☐ 1933 ☐ 1934 ☐ 1935 ☐ 1936 ☐ 1937 ☐ 1938
☐ 1939 ☐ 1940 ☐ 1941 ☐ 1942 ☐ 1943 ☐ 1944 ☐ 1945 ☐ 1946 ☐ 1947
☐ 1948 ☐ 1949 ☐ 1950 ☐ 1951 ☐ 1952 ☐ 1953 ☐ 1954 ☐ 1955 ☐ 1956
☐ 1957 ☐ 1958 ☐ 1959 ☐ 1960 ☐ 1961 ☐ 1962 ☐ 1963 ☐ 1964 ☐ 1965
☐ 1966 ☐ 1967 ☐ 1968 ☐ 1969 ☐ 1970 ☐ 1971 ☐ 1972 ☐ 1973 ☐ 1974
☐ 1975 ☐ 1976 ☐ 1977 ☐ 1978 ☐ 1979 ☐ 1980 ☐ 1981 ☐ 1982 ☐ 1983
☐ 1984 ☐ 1985 ☐ 1986 ☐ 1987 ☐ 1988 ☐ 1989 ☐ 1990 ☐ 1991 ☐ 1992
☐ 1993 ☐ 1994 ☐ 1995 ☐ 1996 ☐ 1997 ☐ 1998 ☐ 1999 ☐ 2000 ☐ 2001
☐ 2002 ☐ 2003 ☐ 2004 ☐ 2005 ☐ 2006 ☐ 2007 ☐ 2008 ☐ 2009 ☐ 2010
☐ 2011 ☐ 2012 ☐ 2013 ☐ 2014 ☐ 2015 ☐ 2016 ☐ 2017 ☐ 2018 ☐ 2019
☐ 2020 ☐ 2021 ☐ 2022 ☐ 2023 ☐ 2024 ☐ 2025 ☐ 2026 ☐ 2027 ☐ 2028
☐ 2029 ☐ 2030 ☐ Other ☐ Don't know

Please specify ________________________________

Have you ever had a head injury that required medical attention
○ Yes
○ No

Type of visit(s)
○ Office visit
○ ER visit
○ Hospitalization
○ Multiple

Year(s)

☐ 1930	☐ 1931	☐ 1932	☐ 1933	☐ 1934	☐ 1935	☐ 1936	☐ 1937	☐ 1938
☐ 1939	☐ 1940	☐ 1941	☐ 1942	☐ 1943	☐ 1944	☐ 1945	☐ 1946	☐ 1947
☐ 1948	☐ 1949	☐ 1950	☐ 1951	☐ 1952	☐ 1953	☐ 1954	☐ 1955	☐ 1956
☐ 1957	☐ 1958	☐ 1959	☐ 1960	☐ 1961	☐ 1962	☐ 1963	☐ 1964	☐ 1965
☐ 1966	☐ 1967	☐ 1968	☐ 1969	☐ 1970	☐ 1971	☐ 1972	☐ 1973	☐ 1974
☐ 1975	☐ 1976	☐ 1977	☐ 1978	☐ 1979	☐ 1980	☐ 1981	☐ 1982	☐ 1983
☐ 1984	☐ 1985	☐ 1986	☐ 1987	☐ 1988	☐ 1989	☐ 1990	☐ 1991	☐ 1992
☐ 1993	☐ 1994	☐ 1995	☐ 1996	☐ 1997	☐ 1998	☐ 1999	☐ 2000	☐ 2001
☐ 2002	☐ 2003	☐ 2004	☐ 2005	☐ 2006	☐ 2007	☐ 2008	☐ 2009	☐ 2010
☐ 2011	☐ 2012	☐ 2013	☐ 2014	☐ 2015	☐ 2016	☐ 2017	☐ 2018	☐ 2019
☐ 2020	☐ 2021	☐ 2022	☐ 2023	☐ 2024	☐ 2025	☐ 2026	☐ 2027	☐ 2028
☐ 2029	☐ 2030	☐ Other	☐ Don't know					

Please specify ______________________________

Cognitive screening

Was the task completed?
◯ Yes
◯ No

Test used
◯ MoCA
◯ Mini Mental
◯ STMS

Reason not performed

☐ Acuity of condition ☐ Cognitive impairment ☐ Dementia ☐ Illiterate ☐ Mental status change
☐ Patient nonverbal ☐ Patient unresponsive ☐ Psychiatric disorder ☐ Totally blind ☐ Totally deaf
☐ Unable to speak primary language ☐ Unstable vital signs ☐ Other

Other - please specify ______________________________

Total Points for MoCA: ______________________________

Total Points for Mini Mental: ______________________________

Total Points for STMS: ______________________________

Interpretation: Within normal limits

Interpretation: Possible cognitive impairment

Depression screening

Was the task completed?

◯ Yes
◯ No

Reason not performed

☐ Acuity of condition ☐ Cognitive impairment ☐ Dementia ☐ Illiterate ☐ Mental status change
☐ Patient nonverbal ☐ Patient unresponsive ☐ Psychiatric disorder ☐ Totally blind ☐ Totally deaf
☐ Unable to speak primary language ☐ Unstable vital signs ☐ Other

Test used

◯ Beck
◯ MADRS
◯ GDS

Total points for Beck ______________________________

Total points for MADRS ______________________________

Total points for GDS ______________________________

Interpretation: Screens negative for depression

Interpretation: Screens positive for depression

History

Medical History

Medical History - Neurological
- ☐ Brain tumor
- ☐ Meningitis/Encephalitis
- ☐ Multiple Sclerosis
- ☐ Neuropathy
- ☐ Normal Pressure Hydrocephalus
- ☐ Seizures
- ☐ Stroke
- ☐ Traumatic Brain Injury
- ☐ None of the above

Medical History - Cancer
- ☐ Melanoma
- ☐ Prostate Cancer
- ☐ None of the above

Medical History - Metabolic
- ☐ Diabetes
- ☐ Gout
- ☐ Hypercholesterolemia
- ☐ Hyperthyroidism
- ☐ Hyperuricemia
- ☐ Kidney Stones
- ☐ Vitamin D deficiency
- ☐ None of the above

Medical History - Sleep Related
- ☐ Periodic Leg Movements of Sleep
- ☐ REM Sleep Behavior Disorder
- ☐ Restless Legs Syndrome
- ☐ Sleep Apnea
- ☐ None of the above

Medical History - Cardiovascular
- ☐ Cardiovascular disease
- ☐ Hypertension
- ☐ None of the above

Medical History - Psychiatric
- ☐ Anxiety disorder
- ☐ Bipolar Disorder
- ☐ Depression
- ☐ Schizophrenia
- ☐ None of the above

Other medical history ________________________________

Family History

Family history of Parkinsonism?
- ○ Yes
- ○ No

Affected 1st & 2nd degree relatives
- ☐ Mother/Father
- ☐ Maternal Uncle
- ☐ Maternal Aunt
- ☐ Paternal Uncle
- ☐ Paternal Aunt
- ☐ Son/Daughter

Number of Mother/Father affected
- ○ 1
- ○ 2

Number of Maternal Uncles affected

- ◯ 1
- ◯ 2
- ◯ 3
- ◯ 4
- ◯ 5
- ◯ 6
- ◯ 7
- ◯ 8
- ◯ 9
- ◯ 10
- ◯ 11
- ◯ 12+

Number of Maternal Aunts affected

- ◯ 1
- ◯ 2
- ◯ 3
- ◯ 4
- ◯ 5
- ◯ 6
- ◯ 7
- ◯ 8
- ◯ 9
- ◯ 10
- ◯ 11
- ◯ 12+

Number of Paternal Uncles affected

- ◯ 1
- ◯ 2
- ◯ 3
- ◯ 4
- ◯ 5
- ◯ 6
- ◯ 7
- ◯ 8
- ◯ 9
- ◯ 10
- ◯ 11
- ◯ 12+

Number of Paternal Aunts affected

- ◯ 1
- ◯ 2
- ◯ 3
- ◯ 4
- ◯ 5
- ◯ 6
- ◯ 7
- ◯ 8
- ◯ 9
- ◯ 10
- ◯ 11
- ◯ 12+

Number of Sons/Daughters affected

- ◯ 1
- ◯ 2
- ◯ 3
- ◯ 4
- ◯ 5
- ◯ 6
- ◯ 7
- ◯ 8
- ◯ 9
- ◯ 10
- ◯ 11
- ◯ 12+

Comment ______________________________

Family history of other Tremor
- ◯ Yes
- ◯ No

Affected 1st & 2nd degree relatives
- ☐ Mother/Father
- ☐ Maternal Uncle
- ☐ Maternal Aunt
- ☐ Paternal Uncle
- ☐ Paternal Aunt
- ☐ Son/Daughter

Number of Mother/Father affected
- ◯ 1
- ◯ 2

Number of Maternal Uncles affected
- ◯ 1
- ◯ 2
- ◯ 3
- ◯ 4
- ◯ 5
- ◯ 6
- ◯ 7
- ◯ 8
- ◯ 9
- ◯ 10
- ◯ 11
- ◯ 12+

Number of Maternal Aunts affected
- ◯ 1
- ◯ 2
- ◯ 3
- ◯ 4
- ◯ 5
- ◯ 6
- ◯ 7
- ◯ 8
- ◯ 9
- ◯ 10
- ◯ 11
- ◯ 12+

Number of Paternal Uncles affected
- ◯ 1
- ◯ 2
- ◯ 3
- ◯ 4
- ◯ 5
- ◯ 6
- ◯ 7
- ◯ 8
- ◯ 9
- ◯ 10
- ◯ 11
- ◯ 12+

Number of Paternal Aunts affected
- ◯ 1
- ◯ 2
- ◯ 3
- ◯ 4
- ◯ 5
- ◯ 6
- ◯ 7
- ◯ 8
- ◯ 9
- ◯ 10
- ◯ 11
- ◯ 12+

Number of Sons/Daughters affected

- ◯ 1
- ◯ 2
- ◯ 3
- ◯ 4
- ◯ 5
- ◯ 6
- ◯ 7
- ◯ 8
- ◯ 9
- ◯ 10
- ◯ 11
- ◯ 12+

Comment

____

Family history of Dementia

- ◯ Yes
- ◯ No

Affected 1st & 2nd degree relatives

- ☐ Mother/Father
- ☐ Maternal Uncle
- ☐ Maternal Aunt
- ☐ Paternal Uncle
- ☐ Paternal Aunt
- ☐ Son/Daughter

Number of Mother/Father affected

- ◯ 1
- ◯ 2

Number of Maternal Uncles affected

- ◯ 1
- ◯ 2
- ◯ 3
- ◯ 4
- ◯ 5
- ◯ 6
- ◯ 7
- ◯ 8
- ◯ 9
- ◯ 10
- ◯ 11
- ◯ 12+

Number of Maternal Aunts affected

- ◯ 1
- ◯ 2
- ◯ 3
- ◯ 4
- ◯ 5
- ◯ 6
- ◯ 7
- ◯ 8
- ◯ 9
- ◯ 10
- ◯ 11
- ◯ 12+

Number of Paternal Uncles affected

- ◯ 1
- ◯ 2
- ◯ 3
- ◯ 4
- ◯ 5
- ◯ 6
- ◯ 7
- ◯ 8
- ◯ 9
- ◯ 10
- ◯ 11
- ◯ 12+

Number of Paternal Aunts affected

◯ 1
◯ 2
◯ 3
◯ 4
◯ 5
◯ 6
◯ 7
◯ 8
◯ 9
◯ 10
◯ 11
◯ 12+

Number of Sons/Daughters affected

◯ 1
◯ 2
◯ 3
◯ 4
◯ 5
◯ 6
◯ 7
◯ 8
◯ 9
◯ 10
◯ 11
◯ 12+

Comment ________________________________

Other Family history? ________________________________

Surgical History

Oophorectomy?

◯ Yes
◯ No
◯ Not Applicable

History of present illness - Initial

Initial Motor Symptom(s)

Initial Motor symptom(s) - year of onset

◯ Don't know
◯ 1930
◯ 1931
◯ 1932
◯ 1933
◯ 1934
◯ 1935
◯ 1936
◯ 1937
◯ 1938
◯ 1939
◯ 1940
◯ 1941
◯ 1942
◯ 1943
◯ 1944
◯ 1945
◯ 1946
◯ 1947
◯ 1948
◯ 1949
◯ 1950
◯ 1951
◯ 1952
◯ 1953
◯ 1954
◯ 1955
◯ 1956
◯ 1957
◯ 1958
◯ 1959
◯ 1960
◯ 1961
◯ 1962
◯ 1963
◯ 1964
◯ 1965
◯ 1966
◯ 1967
◯ 1968
◯ 1969
◯ 1970
◯ 1971
◯ 1972
◯ 1973
◯ 1974
◯ 1975
◯ 1976
◯ 1977
◯ 1978
◯ 1979
◯ 1980
◯ 1981
◯ 1982
◯ 1983
◯ 1984
◯ 1985
◯ 1986
◯ 1987
◯ 1988
◯ 1989
◯ 1990
◯ 1991
◯ 1992
◯ 1993
◯ 1994
◯ 1995
◯ 1996
◯ 1997
◯ 1998

◯ 1999
◯ 2000
◯ 2001
◯ 2002
◯ 2003
◯ 2004
◯ 2005
◯ 2006
◯ 2007
◯ 2008
◯ 2009
◯ 2010
◯ 2011
◯ 2012
◯ 2013
◯ 2014
◯ 2015
◯ 2016
◯ 2017
◯ 2018
◯ 2019
◯ 2020
◯ 2021
◯ 2022
◯ 2023
◯ 2024
◯ 2025
◯ 2026
◯ 2027
◯ 2028
◯ 2029
◯ 2030

Initial Motor Symptom(s)

☐ Resting tremor
☐ Postural/action tremor
☐ Rigidity
☐ Bradykinesia - difficulty arising from a low chair/turning in bed
☐ Bradykinesia - dysarthria/hypophonia
☐ Bradykinesia - sialorrhea
☐ Bradykinesia - masking
☐ Bradykinesia - reduced dexterity
☐ Bradykinesia - micrographia
☐ Bradykinesia - stooped posture
☐ Bradykinesia - reduced arm swing
☐ Bradykinesia - generalized slowness
☐ Freezing
☐ Falls
☐ Gait Disorder
☐ Other

Please specify ______________________________

Tremor - predominant side

◯ Left
◯ Right
◯ No predominant side

Tremor - location(s)

☐ Head
☐ Face
☐ Lips
☐ Chin
☐ Right hand
☐ Left hand
☐ Right foot
☐ Left foot

Rigidity - predominant side

◯ Left
◯ Right
◯ No predominant side

Bradykinesia - predominant side

○ Left
○ Right
○ No predominant side

Falls - predominant side

☐ Left
☐ Right
☐ Back
☐ Forward
☐ All
☐ Don't know

Please specify ______________________________

Prior Diagnosis

Year of diagnosis of PD or Parkinsonism

○ Don't know
○ 2010
○ 2011
○ 2012
○ 2013
○ 2014
○ 2015
○ 2016
○ 2017
○ 2018
○ 2019
○ 2020
○ 2021
○ 2022
○ 2023
○ 2024
○ 2025
○ 2026
○ 2027
○ 2028
○ 2029
○ 2030

History of present illness - Current

Current Motor Symptom(s) - Past Month

Current motor symptom(s)
- ☐ None
- ☐ Resting tremor
- ☐ Postural/action tremor
- ☐ Rigidity
- ☐ Bradykinesia - masking
- ☐ Bradykinesia - sialorrhea
- ☐ Bradykinesia - dysarthria/hypophonia
- ☐ Bradykinesia - reduced dexterity
- ☐ Bradykinesia - micrographia
- ☐ Bradykinesia - difficulty arising from a low chair/turning in bed
- ☐ Bradykinesia - stooped posture
- ☐ Bradykinesia - reduced arm swing
- ☐ Bradykinesia - generalized slowness
- ☐ Gait Disorder
- ☐ Falls
- ☐ Freezing
- ☐ Dyskinesias
- ☐ Motor Fluctuation
- ☐ Other

Please specify ______________________________

Tremor - predominant side
- ◯ Left
- ◯ Right
- ◯ No predominant side

Tremor - location(s)
- ☐ Head
- ☐ Face
- ☐ Lips
- ☐ Chin
- ☐ Voice
- ☐ Right hand
- ☐ Left hand
- ☐ Right foot
- ☐ Left foot

Rigidity - predominant side
- ◯ Left
- ◯ Right
- ◯ No predominant side

Bradykinesia - predominant side
- ◯ Left
- ◯ Right
- ◯ No predominant side

Falls - direction
- ☐ Left
- ☐ Right
- ☐ Back
- ☐ Forward
- ☐ All
- ☐ Don't know

Freezing - Year of Onset

- ◯ Don't know
- ◯ 1970
- ◯ 1971
- ◯ 1972
- ◯ 1973
- ◯ 1974
- ◯ 1975
- ◯ 1976
- ◯ 1977
- ◯ 1978
- ◯ 1979
- ◯ 1980
- ◯ 1981
- ◯ 1982
- ◯ 1983
- ◯ 1984
- ◯ 1985
- ◯ 1986
- ◯ 1987
- ◯ 1988
- ◯ 1989
- ◯ 1990
- ◯ 1991
- ◯ 1992
- ◯ 1993
- ◯ 1994
- ◯ 1995
- ◯ 1996
- ◯ 1997
- ◯ 1998
- ◯ 1999
- ◯ 2000
- ◯ 2001
- ◯ 2002
- ◯ 2003
- ◯ 2004
- ◯ 2005
- ◯ 2006
- ◯ 2007
- ◯ 2008
- ◯ 2009
- ◯ 2010
- ◯ 2011
- ◯ 2012
- ◯ 2013
- ◯ 2014
- ◯ 2015
- ◯ 2016
- ◯ 2017
- ◯ 2018
- ◯ 2019
- ◯ 2020
- ◯ 2021
- ◯ 2022
- ◯ 2023
- ◯ 2024
- ◯ 2025
- ◯ 2026
- ◯ 2027
- ◯ 2028
- ◯ 2029
- ◯ 2030

Dyskinesias - Year of Onset

- ◯ Don't know
- ◯ 1970
- ◯ 1971
- ◯ 1972
- ◯ 1973
- ◯ 1974
- ◯ 1975
- ◯ 1976
- ◯ 1977
- ◯ 1978
- ◯ 1979
- ◯ 1980
- ◯ 1981
- ◯ 1982
- ◯ 1983
- ◯ 1984
- ◯ 1985
- ◯ 1986
- ◯ 1987
- ◯ 1988
- ◯ 1989
- ◯ 1990
- ◯ 1991
- ◯ 1992
- ◯ 1993
- ◯ 1994
- ◯ 1995
- ◯ 1996
- ◯ 1997
- ◯ 1998
- ◯ 1999
- ◯ 2000
- ◯ 2001
- ◯ 2002
- ◯ 2003
- ◯ 2004
- ◯ 2005
- ◯ 2006
- ◯ 2007
- ◯ 2008
- ◯ 2009
- ◯ 2010
- ◯ 2011
- ◯ 2012
- ◯ 2013
- ◯ 2014
- ◯ 2015
- ◯ 2016
- ◯ 2017
- ◯ 2018
- ◯ 2019
- ◯ 2020
- ◯ 2021
- ◯ 2022
- ◯ 2023
- ◯ 2024
- ◯ 2025
- ◯ 2026
- ◯ 2027
- ◯ 2028
- ◯ 2029
- ◯ 2030

Motor Fluctuation - Year of Onset

◯ Don't know
◯ 1970
◯ 1971
◯ 1972
◯ 1973
◯ 1974
◯ 1975
◯ 1976
◯ 1977
◯ 1978
◯ 1979
◯ 1980
◯ 1981
◯ 1982
◯ 1983
◯ 1984
◯ 1985
◯ 1986
◯ 1987
◯ 1988
◯ 1989
◯ 1990
◯ 1991
◯ 1992
◯ 1993
◯ 1994
◯ 1995
◯ 1996
◯ 1997
◯ 1998
◯ 1999
◯ 2000
◯ 2001
◯ 2002
◯ 2003
◯ 2004
◯ 2005
◯ 2006
◯ 2007
◯ 2008
◯ 2009
◯ 2010
◯ 2011
◯ 2012
◯ 2013
◯ 2014
◯ 2015
◯ 2016
◯ 2017
◯ 2018
◯ 2019
◯ 2020
◯ 2021
◯ 2022
◯ 2023
◯ 2024
◯ 2025
◯ 2026
◯ 2027
◯ 2028
◯ 2029
◯ 2030

Dyskinesias - type(s)
- ☐ Peak dose
- ☐ Biphasic
- ☐ Continuous
- ☐ End of dose
- ☐ Don't know

Current Non Motor Symptom(s) - Past Month

Current non motor symptom(s)
- ☐ None
- ☐ Cognitive impairment
- ☐ Psychosis
- ☐ Compulsions/impulse control disorders
- ☐ Depression
- ☐ Fatigue
- ☐ REM sleep behavior disorder
- ☐ Restless leg syndrome
- ☐ Periodic leg movements of sleep
- ☐ Anosmia
- ☐ Excessive daytime sleepiness NOS
- ☐ Insomnia NOS
- ☐ Sleep apnea
- ☐ Unexplained weight loss
- ☐ Orthostatism
- ☐ Anhidrosis
- ☐ Hyperhydrosis
- ☐ Seborrhea
- ☐ Syncope
- ☐ Dysphagia
- ☐ Constipation
- ☐ Fecal incontinence
- ☐ Urinary incontinence
- ☐ Pain
- ☐ Other

Please Specify ______________________________

Compulsions/impulse control disorders - type
- ☐ Gambling
- ☐ Eating
- ☐ Hobbying
- ☐ Hypersexuality
- ☐ Shopping/spending
- ☐ Other

Course of Symptoms during the last year

Course of symptoms (overall)
- ○ Progressing
- ○ Stable
- ○ Improved
- ○ Other
- ○ N/A
- ○ Don't know

Please Specify ______________________________

History of present illness - Brain Imaging

Brain Imaging

CT head

◯ Yes
◯ No

CT - year of most recent study

- ◯ Don't know
- ◯ 1970
- ◯ 1971
- ◯ 1972
- ◯ 1973
- ◯ 1974
- ◯ 1975
- ◯ 1976
- ◯ 1977
- ◯ 1978
- ◯ 1979
- ◯ 1980
- ◯ 1981
- ◯ 1982
- ◯ 1983
- ◯ 1984
- ◯ 1985
- ◯ 1986
- ◯ 1987
- ◯ 1988
- ◯ 1989
- ◯ 1990
- ◯ 1991
- ◯ 1992
- ◯ 1993
- ◯ 1994
- ◯ 1995
- ◯ 1996
- ◯ 1997
- ◯ 1998
- ◯ 1999
- ◯ 2000
- ◯ 2001
- ◯ 2002
- ◯ 2003
- ◯ 2004
- ◯ 2005
- ◯ 2006
- ◯ 2007
- ◯ 2008
- ◯ 2009
- ◯ 2010
- ◯ 2011
- ◯ 2012
- ◯ 2013
- ◯ 2014
- ◯ 2015
- ◯ 2016
- ◯ 2017
- ◯ 2018
- ◯ 2019
- ◯ 2020
- ◯ 2021
- ◯ 2022
- ◯ 2023
- ◯ 2024
- ◯ 2025
- ◯ 2026
- ◯ 2027
- ◯ 2028
- ◯ 2029
- ◯ 2030

CT - normal/abnormal	☐ Normal<br>☐ Basal ganglia lesion<br>☐ Midbrain lesion<br>☐ Cortical lesion<br>☐ White matter lesion(s)<br>☐ Hydrocephalus (disproportionate)<br>☐ Atrophy<br>☐ Other
Comments - Normal	______________________________
Comments - Basal ganglia lesion	______________________________
Comments - Midbrain lesion	______________________________
Comments - Cortical lesion	______________________________
Comments - White matter lesion(s)	______________________________
Comments - Hydrocephalus (disproportionate)	______________________________
Comments - Atrophy	______________________________
Other - Please specify	______________________________
MRI brain imaging	○ Yes<br>○ No

MRI - year of most recent study

◯ Don't know
◯ 1980
◯ 1981
◯ 1982
◯ 1983
◯ 1984
◯ 1985
◯ 1986
◯ 1987
◯ 1988
◯ 1989
◯ 1990
◯ 1991
◯ 1992
◯ 1993
◯ 1994
◯ 1995
◯ 1996
◯ 1997
◯ 1998
◯ 1999
◯ 2000
◯ 2001
◯ 2002
◯ 2003
◯ 2004
◯ 2005
◯ 2006
◯ 2007
◯ 2008
◯ 2009
◯ 2010
◯ 2011
◯ 2012
◯ 2013
◯ 2014
◯ 2015
◯ 2016
◯ 2017
◯ 2018
◯ 2019
◯ 2020
◯ 2021
◯ 2022
◯ 2023
◯ 2024
◯ 2025
◯ 2026
◯ 2027
◯ 2028
◯ 2029
◯ 2030

MRI - normal/abnormal

☐ Normal
☐ White matter lesion(s)
☐ Hydrocephalus (disproportionate)
☐ Atrophy
☐ Other

Please select:

☐ Basal
☐ Midbrain
☐ Cortical

Please specify

____

DaTscan

◯ Yes
◯ No

DaTscan - year of most recent study

- ◯ Don't know
- ◯ 2000
- ◯ 2001
- ◯ 2002
- ◯ 2003
- ◯ 2004
- ◯ 2005
- ◯ 2006
- ◯ 2007
- ◯ 2008
- ◯ 2009
- ◯ 2010
- ◯ 2011
- ◯ 2012
- ◯ 2013
- ◯ 2014
- ◯ 2015
- ◯ 2016
- ◯ 2017
- ◯ 2018
- ◯ 2019
- ◯ 2020
- ◯ 2021
- ◯ 2022
- ◯ 2023
- ◯ 2024
- ◯ 2025
- ◯ 2026
- ◯ 2027
- ◯ 2028
- ◯ 2029
- ◯ 2030

DaTscan - normal/abnormal

- ☐ Normal
- ☐ Reduced uptake (right striatum)
- ☐ Reduced uptake (left striatum)
- ☐ Reduced uptake (right caudate)
- ☐ Reduced uptake (left putamen)
- ☐ Reduced uptake (right putamen)
- ☐ Reduced uptake (left caudate)

History of present illness Rx Current - PD Surgical Treatment

PD Surgical Treatment

PD surgical treatment?
- ◯ Yes
- ◯ No

Type(s) of PD surgical procedures
- ☐ DBS
- ☐ Thalamotomy
- ☐ Pallidotomy
- ☐ Other

DBS locations
- ☐ Right STN
- ☐ Left STN
- ☐ Right GPI
- ☐ Left GPI
- ☐ Right PPN
- ☐ Left PPN
- ☐ Other(specify)

Please specify ______________________________

DBS right STN year(s) of surgery(ies)

☐ 1990 ☐ 1991 ☐ 1992 ☐ 1993 ☐ 1994 ☐ 1995 ☐ 1996 ☐ 1997 ☐ 1998
☐ 1999 ☐ 2000 ☐ 2001 ☐ 2002 ☐ 2003 ☐ 2004 ☐ 2005 ☐ 2006 ☐ 2007
☐ 2008 ☐ 2009 ☐ 2010 ☐ 2011 ☐ 2012 ☐ 2013 ☐ 2014 ☐ 2015 ☐ 2016
☐ 2017 ☐ 2018 ☐ 2019 ☐ 2020 ☐ 2021 ☐ 2022 ☐ 2023 ☐ 2024 ☐ 2025
☐ 2026 ☐ 2027 ☐ 2028 ☐ 2029 ☐ 2030 ☐ Other ☐ Don't know

Please specify ______________________________

DBS left STN year(s) of surgery(ies)

☐ 1990 ☐ 1991 ☐ 1992 ☐ 1993 ☐ 1994 ☐ 1995 ☐ 1996 ☐ 1997 ☐ 1998
☐ 1999 ☐ 2000 ☐ 2001 ☐ 2002 ☐ 2003 ☐ 2004 ☐ 2005 ☐ 2006 ☐ 2007
☐ 2008 ☐ 2009 ☐ 2010 ☐ 2011 ☐ 2012 ☐ 2013 ☐ 2014 ☐ 2015 ☐ 2016
☐ 2017 ☐ 2018 ☐ 2019 ☐ 2020 ☐ 2021 ☐ 2022 ☐ 2023 ☐ 2024 ☐ 2025
☐ 2026 ☐ 2027 ☐ 2028 ☐ 2029 ☐ 2030 ☐ Other ☐ Don't know

Please specify ______________________________

DBS right GPI year(s) of surgery(ies)

☐ 1990 ☐ 1991 ☐ 1992 ☐ 1993 ☐ 1994 ☐ 1995 ☐ 1996 ☐ 1997 ☐ 1998
☐ 1999 ☐ 2000 ☐ 2001 ☐ 2002 ☐ 2003 ☐ 2004 ☐ 2005 ☐ 2006 ☐ 2007
☐ 2008 ☐ 2009 ☐ 2010 ☐ 2011 ☐ 2012 ☐ 2013 ☐ 2014 ☐ 2015 ☐ 2016
☐ 2017 ☐ 2018 ☐ 2019 ☐ 2020 ☐ 2021 ☐ 2022 ☐ 2023 ☐ 2024 ☐ 2025
☐ 2026 ☐ 2027 ☐ 2028 ☐ 2029 ☐ 2030 ☐ Other ☐ Don't know

Please specify ________________________________

DBS left GPI year(s) of surgery(ies)

☐ 1990 ☐ 1991 ☐ 1992 ☐ 1993 ☐ 1994 ☐ 1995 ☐ 1996 ☐ 1997 ☐ 1998
☐ 1999 ☐ 2000 ☐ 2001 ☐ 2002 ☐ 2003 ☐ 2004 ☐ 2005 ☐ 2006 ☐ 2007
☐ 2008 ☐ 2009 ☐ 2010 ☐ 2011 ☐ 2012 ☐ 2013 ☐ 2014 ☐ 2015 ☐ 2016
☐ 2017 ☐ 2018 ☐ 2019 ☐ 2020 ☐ 2021 ☐ 2022 ☐ 2023 ☐ 2024 ☐ 2025
☐ 2026 ☐ 2027 ☐ 2028 ☐ 2029 ☐ 2030 ☐ Other ☐ Don't know

Please specify ________________________________

DBS right PPN year(s) of surgery(ies)

☐ 1990 ☐ 1991 ☐ 1992 ☐ 1993 ☐ 1994 ☐ 1995 ☐ 1996 ☐ 1997 ☐ 1998
☐ 1999 ☐ 2000 ☐ 2001 ☐ 2002 ☐ 2003 ☐ 2004 ☐ 2005 ☐ 2006 ☐ 2007
☐ 2008 ☐ 2009 ☐ 2010 ☐ 2011 ☐ 2012 ☐ 2013 ☐ 2014 ☐ 2015 ☐ 2016
☐ 2017 ☐ 2018 ☐ 2019 ☐ 2020 ☐ 2021 ☐ 2022 ☐ 2023 ☐ 2024 ☐ 2025
☐ 2026 ☐ 2027 ☐ 2028 ☐ 2029 ☐ 2030 ☐ Other ☐ Don't know

Please specify ______________________________

DBS left PPN year(s) of surgery(ies)

☐ 1990 ☐ 1991 ☐ 1992 ☐ 1993 ☐ 1994 ☐ 1995 ☐ 1996 ☐ 1997 ☐ 1998
☐ 1999 ☐ 2000 ☐ 2001 ☐ 2002 ☐ 2003 ☐ 2004 ☐ 2005 ☐ 2006 ☐ 2007
☐ 2008 ☐ 2009 ☐ 2010 ☐ 2011 ☐ 2012 ☐ 2013 ☐ 2014 ☐ 2015 ☐ 2016
☐ 2017 ☐ 2018 ☐ 2019 ☐ 2020 ☐ 2021 ☐ 2022 ☐ 2023 ☐ 2024 ☐ 2025
☐ 2026 ☐ 2027 ☐ 2028 ☐ 2029 ☐ 2030 ☐ Other ☐ Don't know

Please specify ______________________________

DBS other year(s) of surgery(ies)

☐ 1990 ☐ 1991 ☐ 1992 ☐ 1993 ☐ 1994 ☐ 1995 ☐ 1996 ☐ 1997 ☐ 1998
☐ 1999 ☐ 2000 ☐ 2001 ☐ 2002 ☐ 2003 ☐ 2004 ☐ 2005 ☐ 2006 ☐ 2007
☐ 2008 ☐ 2009 ☐ 2010 ☐ 2011 ☐ 2012 ☐ 2013 ☐ 2014 ☐ 2015 ☐ 2016
☐ 2017 ☐ 2018 ☐ 2019 ☐ 2020 ☐ 2021 ☐ 2022 ☐ 2023 ☐ 2024 ☐ 2025
☐ 2026 ☐ 2027 ☐ 2028 ☐ 2029 ☐ 2030 ☐ Other ☐ Don't know

Please specify ______________________________

Thalamotomy locations

◯ Right
◯ Left
◯ Bilateral

Thalamotomy right year(s) of surgery(ies)

☐ 1990 ☐ 1991 ☐ 1992 ☐ 1993 ☐ 1994 ☐ 1995 ☐ 1996 ☐ 1997 ☐ 1998
☐ 1999 ☐ 2000 ☐ 2001 ☐ 2002 ☐ 2003 ☐ 2004 ☐ 2005 ☐ 2006 ☐ 2007
☐ 2008 ☐ 2009 ☐ 2010 ☐ 2011 ☐ 2012 ☐ 2013 ☐ 2014 ☐ 2015 ☐ 2016
☐ 2017 ☐ 2018 ☐ 2019 ☐ 2020 ☐ 2021 ☐ 2022 ☐ 2023 ☐ 2024 ☐ 2025
☐ 2026 ☐ 2027 ☐ 2028 ☐ 2029 ☐ 2030 ☐ Other ☐ Don't know

Please specify ______________________________

Thalamotomy left year(s) of surgery(ies)

☐ 1990 ☐ 1991 ☐ 1992 ☐ 1993 ☐ 1994 ☐ 1995 ☐ 1996 ☐ 1997 ☐ 1998
☐ 1999 ☐ 2000 ☐ 2001 ☐ 2002 ☐ 2003 ☐ 2004 ☐ 2005 ☐ 2006 ☐ 2007
☐ 2008 ☐ 2009 ☐ 2010 ☐ 2011 ☐ 2012 ☐ 2013 ☐ 2014 ☐ 2015 ☐ 2016
☐ 2017 ☐ 2018 ☐ 2019 ☐ 2020 ☐ 2021 ☐ 2022 ☐ 2023 ☐ 2024 ☐ 2025
☐ 2026 ☐ 2027 ☐ 2028 ☐ 2029 ☐ 2030 ☐ Other ☐ Don't know

Please specify __________________________________

Thalamotomy bilateral year(s) of surgery(ies)

☐ 1990 ☐ 1991 ☐ 1992 ☐ 1993 ☐ 1994 ☐ 1995 ☐ 1996 ☐ 1997 ☐ 1998
☐ 1999 ☐ 2000 ☐ 2001 ☐ 2002 ☐ 2003 ☐ 2004 ☐ 2005 ☐ 2006 ☐ 2007
☐ 2008 ☐ 2009 ☐ 2010 ☐ 2011 ☐ 2012 ☐ 2013 ☐ 2014 ☐ 2015 ☐ 2016
☐ 2017 ☐ 2018 ☐ 2019 ☐ 2020 ☐ 2021 ☐ 2022 ☐ 2023 ☐ 2024 ☐ 2025
☐ 2026 ☐ 2027 ☐ 2028 ☐ 2029 ☐ 2030 ☐ Other ☐ Don't know

Please specify __________________________________

Pallidotomy locations

○ Right
○ Left
○ Bilateral

Pallidotomy right year(s) of surgery(ies)

☐ 1990 ☐ 1991 ☐ 1992 ☐ 1993 ☐ 1994 ☐ 1995 ☐ 1996 ☐ 1997 ☐ 1998
☐ 1999 ☐ 2000 ☐ 2001 ☐ 2002 ☐ 2003 ☐ 2004 ☐ 2005 ☐ 2006 ☐ 2007
☐ 2008 ☐ 2009 ☐ 2010 ☐ 2011 ☐ 2012 ☐ 2013 ☐ 2014 ☐ 2015 ☐ 2016
☐ 2017 ☐ 2018 ☐ 2019 ☐ 2020 ☐ 2021 ☐ 2022 ☐ 2023 ☐ 2024 ☐ 2025
☐ 2026 ☐ 2027 ☐ 2028 ☐ 2029 ☐ 2030 ☐ Other ☐ Don't know

Please specify ________________________________

Pallidotomy left year(s) of surgery(ies)

☐ 1990 ☐ 1991 ☐ 1992 ☐ 1993 ☐ 1994 ☐ 1995 ☐ 1996 ☐ 1997 ☐ 1998
☐ 1999 ☐ 2000 ☐ 2001 ☐ 2002 ☐ 2003 ☐ 2004 ☐ 2005 ☐ 2006 ☐ 2007
☐ 2008 ☐ 2009 ☐ 2010 ☐ 2011 ☐ 2012 ☐ 2013 ☐ 2014 ☐ 2015 ☐ 2016
☐ 2017 ☐ 2018 ☐ 2019 ☐ 2020 ☐ 2021 ☐ 2022 ☐ 2023 ☐ 2024 ☐ 2025
☐ 2026 ☐ 2027 ☐ 2028 ☐ 2029 ☐ 2030 ☐ Other ☐ Don't know

Please specify ________________________________

Pallidotomy bilateral year(s) of surgery(ies)

☐ 1990 ☐ 1991 ☐ 1992 ☐ 1993 ☐ 1994 ☐ 1995 ☐ 1996 ☐ 1997 ☐ 1998
☐ 1999 ☐ 2000 ☐ 2001 ☐ 2002 ☐ 2003 ☐ 2004 ☐ 2005 ☐ 2006 ☐ 2007
☐ 2008 ☐ 2009 ☐ 2010 ☐ 2011 ☐ 2012 ☐ 2013 ☐ 2014 ☐ 2015 ☐ 2016
☐ 2017 ☐ 2018 ☐ 2019 ☐ 2020 ☐ 2021 ☐ 2022 ☐ 2023 ☐ 2024 ☐ 2025
☐ 2026 ☐ 2027 ☐ 2028 ☐ 2029 ☐ 2030 ☐ Other ☐ Don't know

Please specify ______________________________

Motor Assessment

Handedness

Dominant Hand

◯ Right
◯ Left

UPDRS Part III - (Motor Function)

Was the task completed?

◯ Yes
◯ No

Reason not performed

☐ Acuity of condition ☐ Cognitive impairment ☐ Dementia ☐ Illiterate ☐ Mental status change
☐ Patient nonverbal ☐ Patient unresponsive ☐ Psychiatric disorder ☐ Totally blind ☐ Totally deaf
☐ Unable to speak primary language ☐ Unstable vital signs ☐ Other

Motor function test performed

◯ MDS
◯ UPDRS

Do you wish to document the complete test or only the final score?

◯ Document complete test
◯ Final Score only

If the patient is taking carbidopa/levodopa, is the patient in On or Off state?

◯ Off
◯ On
◯ Partly on
◯ N/A
◯ Unknown

Speech

◯ 0 - Normal
◯ 1 - Slight loss of expression, diction and/or volume
◯ 2 - Monotone, slurred but understandable, moderately impaired
◯ 3 - Marked impairment, difficult to understand
◯ 4 - Unintelligible
◯ nt - Not Testable

Facial expression

◯ 0 - Normal
◯ 1 - Minimal hypomimia, could be normal "Poker Face"
◯ 2 - Slight but definitely abnormal diminution of facial expression
◯ 3 - Moderate hypomimia, lips parted some of the time
◯ 4 - Masked or fixed facies with severe or complete loss of facial expression, lips parted 1/4 inch or more
◯ nt - Not Testable

Tremor at rest of FACE, LIPS, CHIN

◯ 0 - Absent
◯ 1 - Slight and infrequently present
◯ 2 - Mild in amplitude and persistent. Or moderate in amplitude, but only intermittently present
◯ 3 - Moderate in amplitude and present most of the time
◯ 4 - Marked in amplitude and present most of the time
◯ nt - Not Testable

Tremor at rest RIGHT hand

- ◯ 0 - Absent
- ◯ 1 - Slight and infrequently present
- ◯ 2 - Mild in amplitude and persistent. Or moderate in amplitude, but only intermittently present
- ◯ 3 - Moderate in amplitude and present most of the time
- ◯ 4 - Marked in amplitude and present most of the time
- ◯ nt - Not Testable

Tremor at rest LEFT hand

- ◯ 0 - Absent
- ◯ 1 - Slight and infrequently present
- ◯ 2 - Mild in amplitude and persistent. Or moderate in amplitude, but only intermittently present
- ◯ 3 - Moderate in amplitude and present most of the time
- ◯ 4 - Marked in amplitude and present most of the time
- ◯ nt - Not Testable

Tremor at rest RIGHT foot

- ◯ 0 - Absent
- ◯ 1 - Slight and infrequently present
- ◯ 2 - Mild in amplitude and persistent. Or moderate in amplitude, but only intermittently present
- ◯ 3 - Moderate in amplitude and present most of the time
- ◯ 4 - Marked in amplitude and present most of the time
- ◯ nt - Not Testable

Tremor at rest LEFT foot

- ◯ 0 - Absent
- ◯ 1 - Slight and infrequently present
- ◯ 2 - Mild in amplitude and persistent. Or moderate in amplitude, but only intermittently present
- ◯ 3 - Moderate in amplitude and present most of the time
- ◯ 4 - Marked in amplitude and present most of the time
- ◯ nt - Not Testable

Action or postural tremor of RIGHT hand

- ◯ 0 - Absent
- ◯ 1 - Slight, present with action
- ◯ 2 - Moderate in amplitude, present with action
- ◯ 3 - Moderate in amplitude with posture holding as well as action
- ◯ 4 - Marked in amplitude, interferes with feeding
- ◯ nt - Not Testable

Action or postural tremor of LEFT hand

- ◯ 0 - Absent
- ◯ 1 - Slight, present with action
- ◯ 2 - Moderate in amplitude, present with action
- ◯ 3 - Moderate in amplitude with posture holding as well as action
- ◯ 4 - Marked in amplitude, interferes with feeding
- ◯ nt - Not Testable

Rigidity NECK

- ◯ 0 - Absent
- ◯ 1 - Slight or detectable only when activated by other movements
- ◯ 2 - Mild to moderate
- ◯ 3 - Marked, but full range of motion easily achieved
- ◯ 4 - Severe, range of motion achieved with difficulty
- ◯ nt - Not Testable

Rigidity RIGHT upper extremity

- ◯ 0 - Absent
- ◯ 1 - Slight or detectable only when activated by mirror or other movements
- ◯ 2 - Mild to moderate
- ◯ 3 - Marked, but full range of motion easily achieved
- ◯ 4 - Severe, range of motion achieved with difficulty
- ◯ nt - Not Testable

Rigidity LEFT upper extremity

- ◯ 0 - Absent
- ◯ 1 - Slight or detectable only when activated by mirror or other movements
- ◯ 2 - Mild to moderate
- ◯ 3 - Marked, but full range of motion easily achieved
- ◯ 4 - Severe, range of motion achieved with difficulty
- ◯ nt - Not Testable

Rigidity RIGHT lower extremity

- ◯ 0 - Absent
- ◯ 1 - Slight or detectable only when activated by mirror or other movements
- ◯ 2 - Mild to moderate
- ◯ 3 - Marked, but full range of motion easily achieved
- ◯ 4 - Severe, range of motion achieved with difficulty
- ◯ nt - Not Testable

Rigidity LEFT lower extremity

- ◯ 0 - Absent
- ◯ 1 - Slight or detectable only when activated by mirror or other movements
- ◯ 2 - Mild to moderate
- ◯ 3 - Marked, but full range of motion easily achieved
- ◯ 4 - Severe, range of motion achieved with difficulty
- ◯ nt - Not Testable

Finger taps RIGHT hand

- ◯ 0 - Normal
- ◯ 1 - Mild slowing and/or reduction in amplitude
- ◯ 2 - Moderately impaired. Definite and early fatiguing. May have occasional arrests in movement
- ◯ 3 - Severely impaired. Frequent hesitation in initiating movements or arrests in ongoing movement
- ◯ 4 - Can barely perform the task
- ◯ nt - Not Testable

Finger taps LEFT hand

- ◯ 0 - Normal
- ◯ 1 - Mild slowing and/or reduction in amplitude
- ◯ 2 - Moderately impaired. Definite and early fatiguing. May have occasional arrests in movement
- ◯ 3 - Severely impaired. Frequent hesitation in initiating movements or arrests in ongoing movement
- ◯ 4 - Can barely perform the task
- ◯ nt - Not Testable

RIGHT hand movements (grips)

- ◯ 0 - Normal
- ◯ 1 - Mild slowing and/or reduction in amplitude
- ◯ 2 - Moderately impaired. Definite and early fatiguing. May have occasional arrests in movement
- ◯ 3 - Severely impaired. Frequent hesitation in initiating movements or arrests in ongoing movement
- ◯ 4 - Can barely perform the task
- ◯ nt - Not Testable

LEFT hand movements (grips)

- ◯ 0 - Normal
- ◯ 1 - Mild slowing and/or reduction in amplitude
- ◯ 2 - Moderately impaired. Definite and early fatiguing. May have occasional arrests in movement
- ◯ 3 - Severely impaired. Frequent hesitation in initiating movements or arrests in ongoing movement
- ◯ 4 - Can barely perform the task
- ◯ nt - Not Testable

Rapid alternating movements of RIGHT hand (pronation-supination)

- ◯ 0 - Normal
- ◯ 1 - Mild slowing and/or reduction in amplitude
- ◯ 2 - Moderately impaired. Definite and early fatiguing. May have occasional arrests in movement
- ◯ 3 - Severely impaired. Frequent hesitation in initiating movements or arrests in ongoing movement
- ◯ 4 - Can barely perform the task
- ◯ nt - Not Testable

Rapid alternating movements of LEFT hand (pronation-supranation)

- ◯ 0 - Normal
- ◯ 1 - Mild slowing and/or reduction in amplitude
- ◯ 2 - Moderately impaired. Definite and early fatiguing. May have occasional arrests in movement
- ◯ 3 - Severely impaired. Frequent hesitation in initiating movements or arrests in ongoing movement
- ◯ 4 - Can barely perform the task
- ◯ nt - Not Testable

RIGHT leg agility

- ◯ 0 - Normal
- ◯ 1 - Mild slowing and/or reduction in amplitude
- ◯ 2 - Moderately impaired. Definite and early fatiguing. May have occasional arrests in movement
- ◯ 3 - Severely impaired. Frequent hesitation in initiating movements or arrests in ongoing movement
- ◯ 4 - Can barely perform the task
- ◯ nt - Not Testable

LEFT leg agility

- ◯ 0 - Normal
- ◯ 1 - Mild slowing and/or reduction in amplitude
- ◯ 2 - Moderately impaired. Definite and early fatiguing. May have occasional arrests in movement
- ◯ 3 - Severely impaired. Frequent hesitation in initiating movements or arrests in ongoing movement
- ◯ 4 - Can barely perform the task
- ◯ nt - Not Testable

Arising from chair

- ◯ 0 - Normal
- ◯ 1 - Slow, or may need more than one attempt
- ◯ 2 - Pushes self up from arms of seat
- ◯ 3 - Tends to fall back and may have to try more than one time, but can get up without help
- ◯ 4 - Unable to arise without help
- ◯ nt - Not Testable

Posture

- ◯ 0 - Normal erect
- ◯ 1 - Not quite erect, slightly stooped posture, could be normal for older person
- ◯ 2 - Moderately stooped posture, definitely abnormal, can be slightly leaning to one side
- ◯ 3 - Severely stooped posture with kyphosis, can be moderately leaning to one side
- ◯ 4 - Marked flexion with extreme abnormality of posture
- ◯ nt - Not Testable

Gait

◯ 0 - Normal
◯ 1 - Walks slowly, may shuffle with short steps, but no festination (hastening steps) or propulsion
◯ 2 - Walks with difficulty, but requires little or no assistance, may have some festination, short steps, or propulsion
◯ 3 - Severe disturbance of gait, requiring assistance
◯ 4 - Cannot walk at all, even with assistance
◯ nt - Not Testable

Postural stability

◯ 0 - Normal
◯ 1 - Retropulsion, but recovers unaided
◯ 2 - Absence of postural response, would fall if not caught by examiner
◯ 3 - Very unstable, tends to lose balance spontaneously
◯ 4 - Unable to stand without assistance
◯ nt - Not Testable

Body bradykinesia and hypokinesia

◯ 0 - None
◯ 1 - Minimal slowness, giving movement a deliberate character, could be normal for some persons. Possibly reduced amplitude.
◯ 2 - Mild degree of slowness and poverty of movement which is definitely abnormal.Alternatively, some reduced amplitude
◯ 3 - Moderate slowness, poverty or small amplitude of movement
◯ 4 - Marked slowness, poverty or small amplitude of movement
◯ nt - Not Testable

Total score UPDRS Classic ________________________________

Maximum score ________________________________

Total score MDS ________________________________

Total score UPDRS Classic ________________________________

Total score - comment ________________________________

Steps To Turn 360 Degrees

Steps to turn 360 degrees

- ◯ Not Testable
- ◯ 1
- ◯ 2
- ◯ 3
- ◯ 4
- ◯ 5
- ◯ 6
- ◯ 7
- ◯ 8
- ◯ 9
- ◯ 10
- ◯ 11
- ◯ 12
- ◯ 13
- ◯ 14
- ◯ 15
- ◯ 16
- ◯ 17
- ◯ 18
- ◯ 19
- ◯ 20
- ◯ 21
- ◯ 22
- ◯ 23
- ◯ 24
- ◯ 25
- ◯ 26
- ◯ 27

UPDRS - ADLs

UPDRS Part II - (Activities of Daily Living)

Was the task completed?
- ◯ Yes
- ◯ No

Reason not performed

☐ Acuity of condition ☐ Cognitive impairment ☐ Dementia ☐ Illiterate ☐ Mental status change
☐ Patient nonverbal ☐ Patient unresponsive ☐ Psychiatric disorder ☐ Totally blind ☐ Totally deaf
☐ Unable to speak primary language ☐ Unstable vital signs ☐ Other

Do you wish to document the complete test or only the final score?
- ◯ Document complete test
- ◯ Final Score only

Speech
- ◯ 0 - Normal
- ◯ 1 - Mildly affected. No difficulty being understood.
- ◯ 2 - Moderately affected. Sometimes asked to repeat statements.
- ◯ 3 - Severely affected. Frequently asked to repeat statements.
- ◯ 4 - Unintelligible most of the time.

Salivation
- ◯ 0 - Normal
- ◯ 1 - Slight but definite excess of saliva in mouth, may have nighttime drooling.
- ◯ 2 - Moderately excessive saliva, may have minimal drooling.
- ◯ 3 - Marked excess of saliva with some drooling.
- ◯ 4 - Marked drooling, requires constant tissue or handkerchief

Swallowing
- ◯ 0 - Normal
- ◯ 1 - Rare choking.
- ◯ 2 - Occasional choking.
- ◯ 3 - Requires soft food.
- ◯ 4 - Requires NG tube or gastrotomy feeding

Handwriting
- ◯ 0 - Normal
- ◯ 1 - Slightly slow or small.
- ◯ 2 - Moderately slow or small. All words are legible.
- ◯ 3 - Severely affected. Not all words are legible.
- ◯ 4 - The majority of words are not legible

Cutting food and handling utensils
- ◯ 0 - Normal
- ◯ 1 - Somewhat slow and clumsy, but no help needed.
- ◯ 2 - Can cut most foods, although clumsy and slow. Some help needed.
- ◯ 3 - Food must be cut by someone, but can still feed slowly.
- ◯ 4 - Needs to be fed.

Dressing
- ◯ 0 - Normal
- ◯ 1 - Somewhat slow, but no help needed.
- ◯ 2 - Occasional assistance with buttoning, getting arms in sleeves.
- ◯ 3 - Considerable help required, but can do some things alone.
- ◯ 4 - Helpless

Hygiene	◯ 0 - Normal<br>◯ 1 - Somewhat slow, but no help needed.<br>◯ 2 - Needs help to shower or bathe, or very slow in hygienic care.<br>◯ 3 - Requires assistance for washing, brushing teeth, combing hair, going to bathroom.<br>◯ 4 - Foley catheter or other mechanical aids
Turning in bed	◯ 0 - Normal<br>◯ 1 - Somewhat slow and clumsy, but no help needed.<br>◯ 2 - Can turn alone or adjust sheets, but with great difficulty.<br>◯ 3 - Can initiate, but not turn or adjust sheets alone.<br>◯ 4 - Helpless
Falling	◯ 0 - None<br>◯ 1 - Rare falling.<br>◯ 2 - Occasionally falls, less than once per day.<br>◯ 3 - Falls an average of once daily.<br>◯ 4 - Falls more than once daily
Freezing when walking	◯ 0 - None<br>◯ 1 - Rare freezing when walking, may have start hesitation.<br>◯ 2 - Occasional freezing when walking.<br>◯ 3 - Frequent freezing. Occasionally falls from freezing.<br>◯ 4 - Frequent falls from freezing
Walking	◯ 0 - Normal<br>◯ 1 - Mild difficulty. May not swing arms or may tend to drag leg.<br>◯ 2 - Moderate difficulty, but requires little or no assistance.<br>◯ 3 - Severe disturbance of walking, requiring assistance.<br>◯ 4 - Cannot walk at all, even with assistance
Tremor	◯ 0 - Absent<br>◯ 1 - Slight and infrequently present.<br>◯ 2 - Moderate, bothersome to patient.<br>◯ 3 - Severe, interferes with many activities.<br>◯ 4 - Marked, interferes with most activities
Sensory complaints related to Parkinsonism	◯ 0 - None<br>◯ 1 - Occasionally has numbness, tingling, or mild aching.<br>◯ 2 - Frequently has numbness, tingling, or aching, not distressing.<br>◯ 3 - Frequent painful sensations.<br>◯ 4 - Excruciating pain
Total score	__________________________________
Total score	__________________________________

H&Y

UPDRS PART V - (HOEHN AND YAHR STAGING)

Hoehn and Yahr Staging

- ◯ 0 - No signs of disease
- ◯ 1 - Unilateral disease
- ◯ 1.5 - Unilateral plus axial involvement
- ◯ 2 - Bilateral disease without impairment of balance
- ◯ 2.5 - Mild bilateral disease with recovery on pull test
- ◯ 3 - Mild to moderate bilateral disease some postural instability physically independent
- ◯ 4 - Severe disability - still able to walk or stand unassisted
- ◯ 5 - Wheelchair bound or bedridden unless aided
- ◯ nt - Not Testable

S&E

UPDRS PART VI - (SCHWAB AND ENGLAND ACTIVITIES OF DAILY LIVING)

Percent

- ◯ 100% - Completely independent. Able to do all chores without slowness, difficulty or impairment.Essentially normal. Unaware of any difficulty.
- ◯ 90% - Completely independent. Able to do all chores with some degree of slowness, difficulty and impairment. Might take twice as long. Beginning to be aware of difficulty.
- ◯ 80% - Completely independent in most chores. Takes twice as long. Conscious of difficulty and slowness
- ◯ 70% - Not completely independent. More difficulty with some chores. Three to four times as long in some. Must spend a large part of the day with chores.
- ◯ 60% - Some dependency. Can do most chores, but exceedingly slowly and with much effort. Errors, some impossible.
- ◯ 50% - More dependent. Help with half, slower, etc. Difficulty with everything.
- ◯ 40% - Very dependent. Can assist with all chores, but few alone.
- ◯ 30% - With effort, now and then does a few chores alone or begins alone. Much help needed.
- ◯ 20% - Nothing alone. Can be a slight help with some chores. Severe invalid.
- ◯ 10% - Totally dependent, helpless. Complete invalid.
- ◯ 0% - Vegetative functions such as swallowing, bladder and bowel functions are not functioning. Bedridden.

Bower Criteria

FIRST DIAGNOSTIC LEVEL

Parkinsonism?
- ◯ Yes
- ◯ No

PRESENCE (OR HISTORY) OF 2 OF 4 CARDINAL SIGNS

Rigidity
- ◯ Yes
- ◯ No
- ◯ Don't know

Bradykinesia
- ◯ Yes
- ◯ No
- ◯ Don't know

Resting Tremor
- ◯ Yes
- ◯ No
- ◯ Don't know

Impaired Postural Reflexes
- ◯ Yes
- ◯ No
- ◯ Don't know

DIFFERENTIAL DIAGNOSIS CHECKLIST

Failed levodopa trial (>1000 mg daily)
- ◯ Yes
- ◯ No

Rapid disease progression to H&Y stage 5 in < 5 years
- ◯ Yes
- ◯ No

Features suggesting a secondary cause of parkinsonism

Abrupt onset, non-progressive course
- ◯ Yes
- ◯ No

Step-wise course / repeated strokes
- ◯ Yes
- ◯ No

Repeated head injury
- ◯ Yes
- ◯ No

Neuroleptic exposure
- ◯ Yes
- ◯ No

Imaging shows: hydrocephalus
- ◯ Yes
- ◯ No

Imaging shows: extensive white matter disease
- ◯ Yes
- ◯ No

Imaging shows: basal ganglia / midbrain tumor
- ◯ Yes
- ◯ No

Imaging shows: basal ganglia / midbrain infarct
- ◯ Yes
- ◯ No

Imaging shows: cerebellar or brainstem atrophy / signal abnormality
◯ Yes
◯ No

Features suggesting a degenerative cause of parkinsonism (other than Parkinson's disease)

Early / prominent dementia
◯ Yes
◯ No

Early / prominent falls
◯ Yes
◯ No

Pseudobulbar signs (2 of: spastic dysarthria, dysphagia, affect)
◯ Yes
◯ No

Dystonia
◯ Yes
◯ No

Myoclonus
◯ Yes
◯ No

Pyramidal signs
◯ Yes
◯ No

Amyotrophy
◯ Yes
◯ No

Neuropathy
◯ Yes
◯ No

Early / prominent dysautonomia
◯ Yes
◯ No

Cerebellar signs
◯ Yes
◯ No

Wide-based shuffling gait
◯ Yes
◯ No

Supranuclear downgaze paresis
◯ Yes
◯ No

Axial > limb rigidity
◯ Yes
◯ No

Unilateral signs or pronounced asymmetry beyond 5 years
◯ Yes
◯ No

Limb apraxia
◯ Yes
◯ No

Alien limb
◯ Yes
◯ No

Mirror movements
◯ Yes
◯ No

Cortical sensory loss
◯ Yes
◯ No

FINAL DIAGNOSIS OF PARKINSONISM

Final Diagnosis of Parkinsonism

- ◯ Parkinson's Disease
- ◯ Parkinson's Disease and Dementia
- ◯ Lewy Body Dementia
- ◯ Drug-Induced Parkinsonism
- ◯ Multiple System Atrophy - C
- ◯ Multiple System Atrophy - P
- ◯ Post-Encephalitic Parkinsonism
- ◯ Cerebrovascular Disease with Parkinsonism Features
- ◯ Progressive Supranuclear Palsy
- ◯ Cortical-basal Syndrome
- ◯ Parkinsonism Unspecified

CLINICAL CHARACTERISTICS OF PARKINSON'S DISEASE

Disease subclassification

- ◯ Tremor predominant
- ◯ Akinetic-rigid predominant
- ◯ Mixed

L-DOPA Response

- ◯ No or minimal improvement on >= 1 gram L-dopa/day
- ◯ More than minimal improvement at any dose
- ◯ Poor response but < 1 gram L-dopa/day
- ◯ Unclear
- ◯ Not Applicable

Response to other dopaminergic (more than minimal)

- ◯ Yes
- ◯ No
- ◯ Not Applicable

DNA

DNA Storage?
◯ Yes
◯ No

Year Stored
◯ 2015
◯ 2016
◯ 2017
◯ 2018
◯ 2019
◯ 2020
◯ 2021
◯ 2022
◯ 2023
◯ 2024
◯ 2025
◯ 2026
◯ 2027
◯ 2028
◯ 2029
◯ 2030

DNA Lab Director's Last Name ________________________________

DNA Lab Director's First Name ________________________________

Candidate Gene Genotyping
◯ Yes
◯ No

gene symbol(s) ________________________________

Whole Genome Genotyping
◯ Yes
◯ No

Whole Exome Sequencing
◯ Yes
◯ No

Whole Genome Sequencing
◯ Yes
◯ No

Vital Status

Annual assessment complete?
◯ Yes
◯ No

Lost to Death?
◯ Yes
◯ No

Year of death
◯ 2015
◯ 2016
◯ 2017
◯ 2018
◯ 2019
◯ 2020
◯ 2021
◯ 2022
◯ 2023
◯ 2024
◯ 2025
◯ 2026
◯ 2027
◯ 2028
◯ 2029
◯ 2030

Brain Autopsy?
◯ Yes
◯ No

Lost to follow-up ?
◯ Yes
◯ No
